# Health promotion research involving older adults in the Nordic countries: a scoping review of doctoral theses

**DOI:** 10.1017/S1463423626101248

**Published:** 2026-05-11

**Authors:** Anna Nivestam, Anne Seneca Terkelsen, Anne Clancy, Johanne Lind, Nina Simonsen

**Affiliations:** 1 Department of Nursing and Integrated Health Sciences, Kristianstad University, Sweden; 2 Center for Shared Decision Making, Lillebaelt Hospital, University Hospital of Southern Denmark, Denmark; 3 Institutt for helse- og omsorgsfag, The Arctic University of Norway, Norway; 4 Independent Scholar, Denmark; 5 Folkhälsan Research Center, Public Health Research Program; Department of Public Health, University of Helsinki, Finland

**Keywords:** Aging, doctoral thesis, health promotion, healthy aging participation, older people

## Abstract

There is an increase in life expectancy in the Nordic countries. Research in health promotion is vital in the fields of aging and health to address present and emerging opportunities and challenges. This scoping review of doctoral theses with a health promotion focus was conducted by the ‘healthy ageing’ research group, which is part of the Nordic Health Promotion Research Network. The overall aim was to explore how research about older adults under the label ‘health promotion’ was undertaken in doctoral theses in a Nordic context, with a specific focus on the theoretical framework, and on participation, and empowerment – the guiding principles for health promotion. A scoping review following the guidelines of The Joanna Briggs Institute was carried out. Relevant databases in Denmark, Finland, Norway, and Sweden were searched for theses published between 2011 and 2021. In total 28 theses were included in the results. The results revealed that the number of theses identified with the word ‘health promotion’ in the title, as a keyword, or in the abstract varied considerably between the Nordic countries. Qualitative and quantitative methods were used, and data was collected mainly from healthy older adults living at home. Theoretical perspectives differed, and definitions of health promotion varied across the theses and were sometimes absent. In conclusion, doctoral theses within the field of healthy aging research could benefit from being more specific about the meaning and use of the concept of health promotion.

## Introduction

Within the Nordic countries, life expectancy is increasing, and the population above the age of 65 years will double in the coming decades (Bodin *et al*., 2020). Age is a well-known determinant of health (Dahlgren and Whitehead, 1991), and with higher age, the risk of diseases and physiological decline increases (Clegg *et al*., 2013; Chang *et al*., 2019). The increased risk of diseases can result in a higher demand for health services, thereby raising societal costs (Bloom *et al*., 2015). However, due to older adults’ knowledge and experience they are a valuable resource for society. Older adults make significant contributions through informal caregiving for relatives (Sahlen *et al*., 2012). Additionally, many older adults remain active in the workforce. Their contributions also extend to volunteer work but also to organizations, and associations, and politics (Vercauteren *et al*., 2024). The World Health Organization’s (WHO) definition of older adults in developed world economies as individuals aged 65 years and above (Eurostat, 2020) was used in this study.

To maintain older adults’ capabilities, increase good quality of life in older age, and tackle challenges that come with increased age, it is important to focus on health promotion, which has the potential to maintain and improve health. The Ottawa Charter for Health Promotion (WHO, 1986) has provided a basis for the development of health promotion (Rootman *et al*., 2001), which, in the Charter, is defined as ‘the process of enabling people to increase control over, and to improve, their health’ (WHO, 1986: 1). Health promotion actions optimize the social, environmental, and economic determinants of health and can positively impact individual and population health (WHO, 1986). Health promotion is regarded as a cornerstone of primary health care, and addressing the health needs of older adults within this framework has been underscored (WHO, 2018). To meet the present challenges with an aging population, it is crucial to increase the knowledge about health promotion research in aging and health.

According to McQueen *et al*. (2007), theories of health promotion often fall short of describing health promotion as a field of study and practice. Additionally, there is a lack of consensus on the benefits of theory-based interventions (Cummins, 2022). Health promotion can thus be considered a ‘fuzzy concept’. However, looking at health as a resource provides the basis for health promotion theory (Clancy, 2010). This perspective emphasizes a positive view of health, which aligns with the concepts of salutogenesis and wellbeing (Haugen and Eriksson, 2021). It can be surmised that several theoretical frameworks can support the practice of health promotion.

Participation and empowerment are two core principles in health promotion (Rootman, 2001). Empowerment focuses on enabling people to gain more power over factors that affect their health (Rootman *et al*., 2001). Participation in health promotion initiatives refers to ‘involving all concerned at all stages of the process’ (Rootman *et al*., 2001: 4). Recent research from the Nordic countries has highlighted the importance of the involvement of ‘users’ in the research process (Blix and Hamran, 2021; Koski and Pihlainen, 2022; Kylén *et al*., 2022; Pedersen *et al*., 2022). Kylén *et al*. (2022) showed in their study, from the perspective of researcher, that the most important reason for involving users in the research process was to make the research relevant for practice. User involvement has the potential to improve the quality of health research and can also empower users of health and social care services (Shippee *et al*., 2015). For example, older adults could be involved in formulating research questions, collecting data, and analyzing data (Iwarsson *et al*., 2019; Kylén *et al*., 2022). A practical example from Denmark shows that user involvement greatly benefits and advances the project (Pedersen *et al*., 2022).

Research focusing on health promotion interventions among older adults has increased significantly over the past years (Chiu *et al*., 2020). Various types of health promotion interventions have been identified in scoping reviews (Duplaga *et al*., 2016; Chiu *et al*., 2020). However, these reviews often lack details on the theories and definitions of health promotion. Since theories play a crucial role in health promotion, research is needed to identify the specific theories and definitions applied in this field. Health promotion is context-dependent, and as the Nordic countries share many similarities in history and legislation (Thualagant *et al*., 2023), it is valuable to explore research focused on health promotion and older adults within the Nordic context. Doctoral theses provide deeper insights into the theories and definitions used in research articles, therefore it is beneficial to examine recent theses rather than peer reviewed scientific articles (Eriksson *et al*., 2020). The doctoral degree is the highest academic qualification. In the Nordic countries, full-time doctoral education spans three to four years, awarding 180 to 240 ECTS (European Credit Transfer and Accumulation System). During this period, a thesis is written, either as a monograph or, more commonly nowadays, as a compilation thesis. A compilation thesis consists of approximately three to five scientific articles summarized in a comprehensive report. This study aimed to explore how research concerning older adults under the label ‘health promotion’ is conducted in doctoral theses within a Nordic context, with a specific focus on theoretical frameworks and the principles of participation and empowerment, which guide health promotion. This knowledge can help identify future research directions and guide researchers in their work.

## Methods

### Design

A scoping review design was used in this study as it is an appropriate method to determine the breadth and volume of research in the area and identify possible knowledge gaps (Munn *et al*., 2018). The review process was inspired by the methodological framework proposed by The Joanna Briggs Institute (JBI) (Peters *et al*., 2020). To guide the process and to increase methodological transparency and the comprehension of our findings, we used the PRISMA-ScR (Preferred Reporting Items for Systematic Reviews and Meta-Analysis extension for Scoping Reviews) (Tricco *et al*., 2018).

### Inclusion criteria

#### Participants

Theses with participants aged 65 years and above were included in the study. Theses focusing on healthcare personnel were excluded. The motivation for including only theses with a focus on the views of older adults was to empower their perspectives.

#### Concept

The focus of our search was to locate doctoral thesis research with the concept ‘health promotion’ and variations of the term (e.g., health promoting, promoting health), to be found in either title, abstract or keywords.

#### Context

Only theses with empirical studies where data collection was conducted in the Nordic countries – Denmark, Finland, Norway, and Sweden – were included in the review.

### Search strategy

The search strategy was planned and conducted by all authors between January to December 2022. All searches were made with guidance from research librarians. Different databases were used in different countries to find eligible theses. In Denmark, there is no longer a joint database for identifying doctoral theses from the different universities. The search was thus limited to searches conducted in databases at three institutions (Aalborg University, The Royal Danish Library and University College Lillebælt), which at that point were the only databases providing information on doctoral theses. In Finland, the searches were carried out in two databases, Finna and Medic. In Norway, the searches were carried out in the databases Nora and Oria. In Sweden, the databases Diva, Libris and SwePub were used to identify theses.

The authors agreed on English and Scandinavian search terms and conducted an initial search in databases relevant to each of the included countries. After the initial search, any considerations regarding the search strategy were discussed and resolved and a final search was conducted. We searched for the concepts; health promot* AND old* OR age* OR aging OR senior* OR elder* and similar terms in the Scandinavian languages. Danish terms: ‘Sundhedsfremme’ and ‘ældre’, Norwegian search terms: Helsefrem* eldre* og aldring, Finnish and Swedish terms: ‘Terveyden edistäminen’/’Terveyttä edistävä’/’Terveydenedist*/‘Hälsofrämjande’ and ‘vanh*’, ‘ikä’/’äldre’.

The search was limited to theses published between 2011 and 2021 as we aimed to incorporate contemporary research that mirrors today’s society. In the Swedish and Finnish databases, additional limitations were set to include only doctoral thesis. Theses published in the Scandinavian languages, Danish, Finnish, Norwegian and Swedish as well as English were considered for inclusion since the authors are proficient in these languages.

### Source of evidence screening and selection

All authors independently reviewed the title and abstract of the theses found in their own country (the authors origin from Denmark, Finland, Norway, or Sweden) and excluded theses that did not meet the inclusion criteria. Subsequently, full-text theses were divided between the authors and screened for relevance to ensure that they met the inclusion criteria. Any questions regarding the included theses were discussed in the author group and resolved.

### Data extraction

Both monographs and compilation theses were found eligible for this study. A compilation thesis normally includes a comprehensive summary and the scientific manuscripts written during the doctoral education. Data was extracted only from the comprehensive summary. Data from all included theses were extracted by a minimum of two authors independently. The extracted data included author, university, year of doctoral defense, aim, research method(s) used, main theory applied and definition of health promotion, the context in which the thesis was conducted, the population studied, data collection method(s) and conclusion(s) of the theses.

### Data analysis and presentation of results

The abstracts of the theses were read several times and the characteristics of the theses: aim/focus, methods, context/population, and conclusions were examined and extracted. To find theories used and definitions of health promotion, the entire thesis was searched for the terms: ‘theory’ and ‘health promotion’. Moreover, we searched for the terms ‘empow*’, and ‘enabl*’ and counted the number of times and in which section of the thesis the terms were mentioned. Thereafter we searched for ‘participant*’, and ‘involv*’ within each thesis to find out if a participatory design was used. Data extracted from the included theses were then described in figures and text. The included theses were summarized based on the aim/focus, methods, context/population, and conclusions as well as theory and how health promotion was defined.

## Results

### Literature search

**Figure 1.**
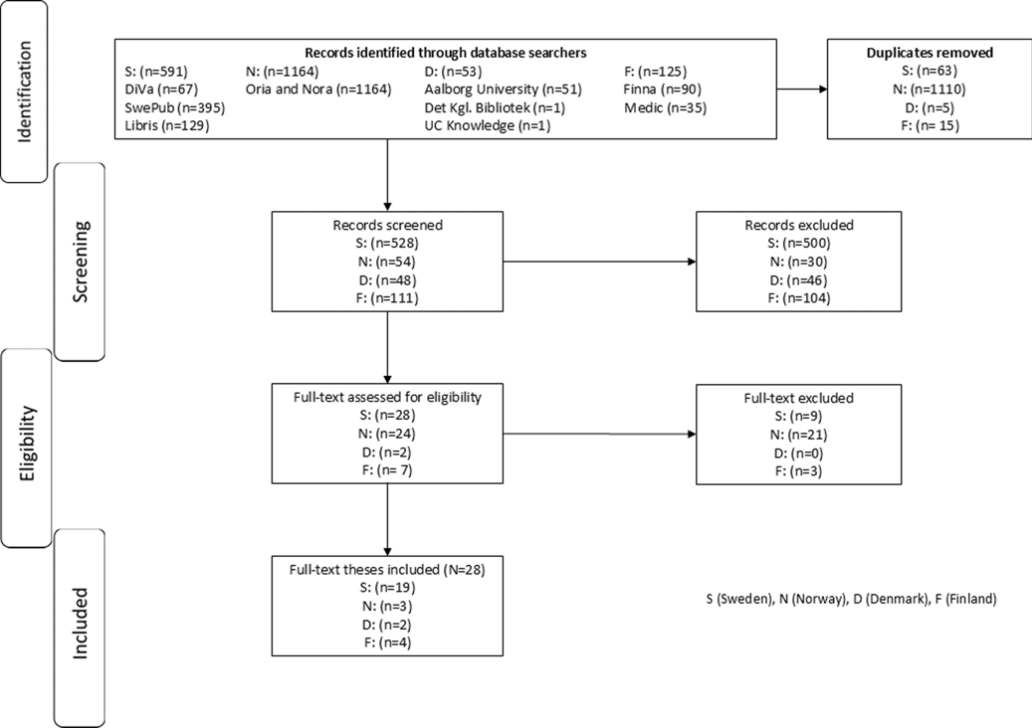
Flow chart, an illustration of the selection process.

The search for relevant theses resulted in a total of 28 theses from Denmark, Finland, Norway and Sweden. See flow chart Figure 1 for an illustration of the selection process.

From the Danish institutions, first a total of 53 theses were identified: University College Lillebælt (*n* = 1), The Royal Danish Library (*n* = 1), and Aalborg University (*n* = 51). No duplicates were identified, but five theses were removed before screening as they were published before 2011. Forty-eight theses were screened by title/abstract, and 46 theses were removed. This left two theses for full-text reading included from Denmark.

In the Finnish databases, first a total of 126 theses were found: Medic (*n* = 35) and Finna (*n* = 91). Fifteen duplicates were removed. One hundred eleven theses were screened by title/abstract, and 104 theses were removed. This left seven theses for full-text reading. Three were excluded, resulting in four included theses from Finland. One of the excluded Finnish theses was included among the Swedish theses as the doctoral studies were conducted at the Nordic School of Public Health, located in Sweden.

In the Norwegian databases Oria and Nora, first a total of 1164 theses were found. Duplicates were removed (*n* = 1110). The remaining 54 theses were screened by title/abstract, and 30 were removed. This left 24 theses for full-text reading. Twenty-one were excluded, resulting in three included theses from Norway.

In the Swedish databases, first a total of 591 theses were found: Libris (*n* = 129), Diva (*n* = 67), and SwePub (*n* = 395). Sixty-three duplicates were removed. The remaining 528 theses were screened by title/abstract, with 500 theses removed. This left 28 theses for full-text reading. Nine were excluded, resulting in 19 included theses from Sweden.

#### Characteristics of theses

The theses were published between 2012 and 2021 and conducted in Denmark at the University of Copenhagen (*n* = 2); in Finland at Aalto University (*n* = 1), Åbo Akademi University (*n* = 1), University of Eastern Finland (*n* = 1), and the University of Turku (*n* = 1); in Norway at the University of Oslo (*n* = 2) and the Norwegian University of Science and Technology (*n* = 1); and in Sweden at the University of Gothenburg (*n* = 6), Luleå University of Technology (*n* = 3), Umeå University (*n* = 3), Karolinska Institutet (*n* = 3), Malmö University (*n* = 1), Linköping University (*n* = 1), and the Nordic School of Public Health (*n* = 2). The Nordic School of Public Health (1959–2014), which was financed by all the Nordic countries (Suominen, 2014), was situated in Sweden and is here included among the Swedish universities. Characteristics of the theses are presented in Table 1. The included theses represented various health sciences disciplines (nursing, health and social work, oral health, public health, rehabilitation, occupational health, neurobiology/neuroscience, and physiology as well as nutrition, exercise, and sports). The fields of technology and engineering were also represented (Table 1).

**Table 1.** Study characteristics of included theses (*n* = 28). Denmark(D), Finland (F), Norway(N) and Sweden(S)

	Author, year, title, university	Aim	Research method(s)	Data collection method(s)	Context	Population	Conclusion
**S**	Arola 2018 *Capturing the experience of health among persons aging in a migration context. Health promotion interventions as means to enable health and occupations in daily life.* Department of Health and Rehabilitation, Institute of Neuroscience and Physiology, Sahlgrenska Academy, University of Gothenburg, Gothenburg, Sweden	The overall aim of this thesis was to increase our understanding of health in everyday life among older persons aging in migration, and to evaluate the outcome of a health promotion intervention on sense of coherence, life satisfaction and engagement in activities.	Qualitative and quantitative	Questionnaires and assessment (sense of coherence, Life satisfaction, engagement in activities of interest), individual interviews, and focus groups	Home suburban district in a middle-sized town in Sweden	Persons aging in Sweden, born in Finland aged ≥ 65 years of age (*n* = 16). Immigrants 70 years og age or older from Finland and Western Balkan (*n* = 131) Health care professionals, social workers, occupational therapist, physiotherapist, nurses, home help professionals (*n* = 16)	Older adults’ perceptions of their abilities, health, and aging shape their everyday health experiences, irrespective of their migration background. Utilizing a group-based, person-centered approach can empower older immigrant populations living at home to recognize and harness their capabilities, thereby sustaining and enhancing their everyday health.
**N**	Aure 2021 *Technology-mediated patient engagement in nutrition care Opportunities for early intervention to support health and independence in old age* Department of Nursing Science, Faculty of Medicine, University of Oslo, Norway	To investigate the feasibility of introducing an app (Appetitus) to prevent undernutrition and to support nutrition care in home care.	Qualitative and quantitative	Individual interviews with older adults and focus group and individual interviews with health care professionals	Home care	25 older adults 25 health care professionals	Support tools such as the Appetitus app, which enhances dietary awareness among older adults, hold significant promise as early interventions to prevent undernutrition.
**S**	Barenfeld 2016 *How to support knowing and doing in promotion of health - Lessons learned from the Promoting Aging Migrants’ Capabilities program* Department of Health and Rehabilitation, Institute of Neuroscience and Physiology, Sahlgrenska Academy at University of Gothenburg, Gothenburg, Sweden	In the context of a researcher-community partnership, this thesis aimed to explore how to support the development and realizing of an adapted health promotion program its benefits, and impact for older persons aging in the context of migration.	Qualitative and quantitative	Focus group discussions, individual interviews, document review, and face-to-face interviews according to a study questionnaire.	Suburban district of medium-sized Swedish city	Health personnel, policymakers and researchers (*n* = 12) and people aged 70–84 years who have participated in the PAMC (Promoting Aging Migrants’ Capabilities) (*n* = 12–14). People aged ≥70 years who migrated to Sweden from Finland or the Western Balkan Region (*n* = 131)	The researcher-community partnership proved successful as an implementation strategy, facilitating the adaptation of the program to overcome barriers to health promotion and yielding tangible benefits in daily life. While both qualitative and quantitative data offered insights, additional studies are required to conclusively assess the program’s effectiveness. Nonetheless, based on observed benefits, the program is recommended for enhancing the capacities of older adults in migration contexts, empowering them to access and utilize health services and opportunities more effectively.
**S**	Behm 2014 *It´s never too late. Health-promotion and disease prevention for very old persons* Institute of Neuroscience and Physiology, Sahlgrenska Academy at University of Gothenburg, Gothenburg, Sweden	To evaluate the effects of health-promoting and disease-preventive interventions on health and frailty in very old community-dwelling persons	Qualitative and quantitative	Focus group, individual interviews, RCT-questionnaires and assessments.	Two urban districts in Gothenburg, Sweden.	459 persons aged 80 years or older and still living at home (*n* = 459)	Health decline in older adults at risk of frailty can be postponed through targeted interventions. These interventions, particularly senior meetings, may motivate health-promoting behaviors by providing holistic information, empowering participants, and focusing on personal needs. The group setting of senior meetings allowed participants to learn from each other, share problems, and find role models, enhancing their understanding and ability to use their own resources. This approach strengthens their role as older adults and encourages engagement in health-promoting activities.
**S**	Björklund 2015 *Temporal Patterns of Daily Occupations and Personal Projects Relevant for Older Persons’ Subjective Health - a Health Promotive Perspective* Division of Health and Rehabilitation, Department of Health Sciences, Luleå University of Technology, Luleå, Sweden	The overall aim of this thesis was to develop knowledge and understanding of temporal patterns of daily occupations and personal projects relevant for older persons’ health and well-being.	Mixed methods	Study 1 and 2: Open time-diaries completed for one week and two health questionnaires: SoC-29 and SF-36. Study 3: Semi-structured individual interviews Study 4: Mixed Methods	Two municipalities in northern Sweden	Older persons aged 70 years or older living in their private urban or rural homes (*n* = 151)	Understanding the temporal patterns of daily occupations provides insights into when older adults engage in various activities throughout the day. This knowledge can enhance opportunities for older adults to remain in their community homes, participate in society, and promote their subjective health.
**S**	Boman 2016 *Inner strength as a health resource among older women* Department of Nursing Umeå University, Sweden	The overall purpose of this thesis was to explore inner strength as a health resource among older women	Qualitative and quantitative	Focus group interviews and questionnaires: The inner strength scale (ISS), The SF12 Health Survey (SF-12) and The Geriatric Depression Scale (GDS).	Åland, Finland	Community-dwelling older women aged 65 years and older living in Åland	The Inner Strength Scale can be used to rate inner strength and guide interventions in beneficial dimensions. Inner strength can also be identified in narratives of older women. Mental ill health shows the strongest association with weakened inner strength among community-dwelling older women.
**D**	Clotworthy 2017 *Empowering the elderly? A qualitative study of municipal home-health visits and everyday rehabilitation* University of Copenhagen, Department of Ethnology, The SAXO Institute, Faculty of Humanities, Denmark	How do the Danish state’s political goals and individualized health policies influence the provision of in-home health services for the elderly, and how do both municipal health professionals and elderly citizens navigate the political discourses that frame their relational encounter?	Qualitative	15 months of ethnographic fieldwork in a Danish municipality. A shadowed and observed everyday work practices of health professionals from three departments and dozens of visits to elderly citizens´ homes as well as during training sessions. Semi-structured interviews with both the professionals and citizens.	Danish municipality	Eight municipal health professionals and five older adults (men/women, aged 67–83 years)	A more caring response to neoliberal conceptualizations about individualism and self-responsibility could generate an alternative form of empowerment, emphasizing collaboration and connectivity. This model of relational power, observed in health encounters, distributes care among various actors in the social environment, fostering freedom and agency with potentially boundless impact on aging citizens, health professionals, and Danish society.
**S**	Fjell 2021 *Preventive home visits among older people: Risk assessment, self-rated health, and experiences of healthy aging* Department of Neurobiology, Care Science and Society Karolinska Institutet, Stockholm, Sweden	The aim of this thesis is to increase the level of knowledge about the content of preventive home visits to older adults who are living at home and about how older adults who live at home perceive the aging process; the overall purpose is to contribute knowledge to help develop risk prevention and health-promotion activities within this population.	Qualitative and quantitative	Questionnaires and focus groups	Three municipalities (small, medium, and large) in Western Norway.	Older persons aged 75 years and older living at home.	The World Health Organization’s focus areas—falls, nutrition, polypharmacy, and cognitive impairment—in preventive home visits for risk identification seem to be relevant. Emphasizing self-rated health in these visits can enhance older adults’ functional ability and support healthy aging. Additionally, focus group interviews reveal that social networks and activities are crucial for maintaining a good life and aging well.
**S**	Forsman 2012 *The Importance of social capital in later life mental health promotion and mental disorder prevention among older adults* Nordic School of Public Health, Gothenburg, Sweden National Institute for Health and Welfare, Finland	The overall aim of the thesis is to examine how mental health and mental well-being can be promoted and how the incidence and prevalence of depressive symptoms and disorders can be prevented among older adults. The specific aims of the included studies are to examine the associations between mental ill-health (depression and psychological distress) and social capital among older adults, as well as to collect and evaluate the effect of psychosocial interventions for the primary prevention of depressive disorders. Another specific aim is to provide a better understanding of how social capital influences the experienced mental well-being among older adults.	Qualitative and quantitative	Systematic review, survey, and focus group interviews	Finland and Sweden	Study 1–3: Older adults aged 65+ years Study 4: Older adults aged 65 + years (focus group interviews) and older adults aged 60–81 years (survey) *N* = 869	There is a need to actively maintain the social networks and interactions of older adults to promote mental health and prevent mental ill-health. It is vital to involve older adults themselves in planning initiatives aimed at enhancing mental health and well-being. Older adults with low social capital are more likely to experience mental ill-health and should have access to initiatives that empower social networking and maintain a rich social life. Additionally, the findings highlight the significant potential of psychosocial interventions in supporting active and healthy aging when appropriately implemented.
**S**	Grundberg 2015 *Mental health promotion among community dwelling seniors with multimorbidity - perspectives of seniors, district nurses and home care assistants* Department of Neurobiology, Care Sciences and Society, Division of Neurogeriatrics, Karolinska Institutet, Stockholm, Sweden and Sophiahemmet University, Stockholm, Sweden	To gain a deeper understanding of how mental health may be promoted among community-dwelling seniors with multiple chronic conditions.	Qualitative	Semi-structured individual interviews and focus group interviews.	Ordinary housing in an urban area in Sweden.	Community-dwelling seniors with multiple chronic conditions, aged 79–96 years 8 *n* = (13) District Nurses from different primary health care centers in the Stockholm region aged 31–83, one retired (*n* = 25) Home care assistants from municipal home help services in one urban district in Sweden aged 21–65 years (*n* = 26)	Seniors with multimorbidity should have the opportunity to describe how multiple chronic conditions affect their life situation. Optimal care can be achieved through continuity, involvement, and health-promoting dialog based on the person’s wishes and needs.
**S**	Gustafsson 2012 *Health-promoting intervention for community-dwelling older adults. Focusing on the concept of frailty and intervention outcome.* Institute of Neuroscience and Physiology, Sahlgrenska Academy, University of Gothenburg, Sweden	The overall aim of this thesis was to increase our understanding of the concept of frailty in relation to older adults, and to review and evaluate outcomes in health-promoting interventions for community-dwelling older adults.	Qualitative and quantitative	Study 1: Systematic literature review-database search Study 2: Focus group discussions Study 3: Questionnaire on frailty indicators: Question about Self-rated Health (SRH) Questionnaire on Activities of Daily Living (ADL) Study 4: Questionnaire on perceived security in ADL	Urban districts in Gothenburg, Sweden	Study 1: Original articles on RCTs concerning community-dwelling persons 65 years or older defined as frail, and with at least one intervention group of participants receiving HPDP interventions Study 2: 21 Health care professionals in 4 groups, aged 26–60 years. Study 3 and 4: 459 older persons aged 80–97 years.	The definition of frailty varies according to different paradigms, highlighting the need for clear definitions in all contexts, particularly in research and health promotion. Health-promoting interventions for older adults at risk of frailty can delay the deterioration of self-rated health and dependence in activities of daily living (ADL) in both the short and long term. Senior meetings appear more effective than a single preventive home visit in delaying deterioration and reducing dependence in ADL. This underscores the potential of the RCT Elderly Persons in the Risk Zone and the importance of further evaluating and developing this health-promoting intervention.
**S**	Kleijberg 2021 *Studio Döbra – Creating spaces for engaging with end-of-life issues and interacting across generations through community-based arts activities.* Dept of Learning, Informatics, Management and Ethics. Karolinska Institute, Stockholm, Sweden	The overall research aim is to investigate the processes and impacts of developing, facilitating, and participating in Studio DöBra. A secondary aim is to investigate the experiences of professionals developing and facilitating initiatives that share characteristics of Studio DöBra.	Qualitative	Meetings, workshops, interviews	Two communities (Skärholmen and Halmstad) in Sweden	Children (9 years old) and older adults (most 80+)	Understanding how community-based intergenerational arts initiatives can be developed and facilitated to engage community organizations, older adults, children, and their parents in end-of-life (EoL) issues and stimulate intergenerational interaction.
**F**	Komulainen 2013 *Oral health promotion among community-dwelling older people* Kuopio Research Centre of Geriatric Care School of Pharmacy, Social Pharmacy Faculty of Health Sciences, University of Eastern Finland, Kuopio, Finland	The aim in this thesis was to study the effect of preventive oral health care intervention among community-dwelling older people. In addition, determinants for the need for preventive oral health care were studied as well as factors associated with oral-self-care and oral hygiene and with the participants who preferred the dentist’s home visit instead of paying a visit to a dental clinic.	Qualitative and quantitative	Data on oral health were obtained in face-to-face interviews and in clinical oral examinations.	Kuopio, Finland	Random sample of persons aged 75 or older living in Kuopio, Finland who resided in community-dwelling and whose oral status was recorded (*n* = 321). At the baseline, there were 165 community-dwelling persons in the oral health intervention group and 156 subjects in the control group.	Preventive oral health care measures can improve the oral health of older adults. However, despite such interventions, the need for preventive oral health care and the prevalence of oral diseases remain high. These findings emphasize the importance of regular dental care by professionals and potential assistance with oral self-care for older adults. Additionally, there is a need to provide oral health services in the home setting.
**F**	Laatikainen 2019 *Environments for healthy and active aging* Aalto University, School of Engineering, Department of Built Environment, Spatial Planning and Transportation Engineering, Finland	The broad aim is to understand how the physical environment in concurrence with individual factors can support healthy and active aging. The possibilities and challenges of online participatory mapping method in health promotion research and among older adults are studied. I also study different physical environmental contexts and how they are associated with the health and physical activity of older adults.	Quantitative	Surveys, maps, SoftGIS surveys.	The Helsinki Metropolitan Area, Finland.	Older adult visitors of Kamppi Service Center, Helsinki. Random sample of adults aged 55 to 74 years. (In one sub-study: children and 15–65-year-olds in order to do comparisons of different age-groups)	The physical environment is fundamental in facilitating health behaviors among older adults, transcending individual preferences and backgrounds. Additionally, a redefined ecological model of physical activity is proposed, emphasizing the significance of contextual factors.
**S**	Larsson 2016 *Promoting social activities and participation among seniors. Exploring and evaluating social and Internet-based occupational therapy interventions* Department of Community Medicine and Rehabilitation, Occupational therapy, Umeå University, Sweden Umeå 2016	The overall aim of this thesis is to increase the knowledge of how Internet-based activities influence seniors’ participation in society, how seniors experience and are influenced by support from a social Internet-based occupational therapy intervention, and how different aspects of this intervention can contribute to healthy aging.	Qualitative and quantitative	Interview, multiple case study, and randomized crossover.	Research group workplace and participants´ home	Community-dwelling, retired seniors without home-care services aged 61 – 89 years.	Social support, technology experiences and accessibility, life-changing events, and meaningful online activities influence motivation to participate in social and internet-based activities. Occupational therapists should consider individual perspectives to tailor interventions, supporting satisfactory participation and enhancing social activities and contacts for seniors.
**F**	Ljungquist 2018 *Caring deeds and habits – for a caritative approach to the act of caring* Faculty of education and welfare studies, Division of Health Sciences, Åbo Akademi University, Finland	The overall aim of the research was to deepen the understanding of ethics and describe the actions and habits that are important in the ‘acts of caring’ for both patients and caregivers in relation to the caritative nursing and ethics of care relevant to the caritative care theory	Qualitative	Study A: Individual interviews. Study B: Questionnaire Study C: Focus group interview (study C)	Several home healthcare contexts	20 home care patients (men/women, aged 55–93 years). Mean age 78 years. 34 carers (29 women and five men, aged 20–61 years). Study C: Five carers. All women, aged 25–64 years.	The caregiver’s inner and outer demeanor, combined with positive character traits such as virtues and moral integrity, grounded in compassion and enacted with a sense of duty, intention, and resilience, are essential for compassionate acts and fostering beneficial caregiving practices.
**S**	Lood 2015 *Discovering the capabilities of aging persons who are born abroad: Crossing norms, moving health promotion forward.* Section for Health and Rehabilitation Institute of Neuroscience and Physiology Sahlgrenska Academy at University of Gothenburg, Sweden.	The overarching aim of this thesis was to explore ethical and empirical points of departure for health promotion in relation to aging persons who have experienced international migration.	Mixed methods	Systematic literature review and meta-analysis, focus group discussions, narrative interviews, and randomized controlled feasibility study.	Community of a low-income, suburban district of a middle-sized Swedish city.	Study II: Qualified social workers, home help professionals, registered nurses, occupational therapists and physiotherapists (*n* = 18) Study III: Persons aged > 65 years of age, migrated from Bosnia and Herzegovina, Croatia, Montenegro and Serbia (*n* = 15) Study IIII: Persons aged > 70 years of age, migrated from Finland, Bosnia and Herzegovina, Croatia, Montenegro and Serbia (*n* = 40)	There is a complex interplay between personal choices and normative power in identifying and reaching health promotion goals in the context of aging and migration. This understanding offers insights into reducing healthcare inequities.
**S**	Mahler 2012 *At holde balance. Betingelser for og perspektiver til forebyggelse af fald blandt gamle mennesker* (Only a Danish title available) Nordic School of Public Health, Göteburg, Sweden	To describe how community-dwelling older adults experience and handle falls, falling and loss of balance. The focus is also on examination of falls as contextual phenomena with the older adults’ experiences. This study will contribute to develop health promotion and fall-prevention to individuals and to the fall-prevention as so.	Qualitative	Individual interviews, focus group interviews, case study, and document analysis.	The older adults were interviewed at home. The health care professionals were interviewed at the home care office.	Community-dwelling older adults aged 75–94 years. Study I: Four women and two men. Study II: Five women. Study III: One woman and one man, two social and health care worker, two social and health care assistants, one nurse. Study IIII: Eight women and four men.	In the future, fall prevention needs to adopt a health promotion approach that contextualizes falling accidents within individuals’ experiences and meanings. This approach should be multifactorial, multidimensional, and existential.
**S**	Mårtensson 2012 *Promoting oral health: Knowledge of periodontal disease and satisfaction with dental care* Malmö University, Department of Oral Public Health, Faculty of Odontology, Malmö, Sweden	The general aims of this thesis were to evaluate if a mass media campaign, aimed as a health promoting campaign, and visits to a specialist clinic in periodontology could increase the knowledge of periodontitis, symptoms, and treatment. A further aim was to analyze expectations and satisfaction with care among patients referred for comprehensive treatment to specialist clinics.	Quantitative	Questionnaires	Sweden (randomly sampled from the population register).	50–75 years old people in Sweden.	There was enhanced knowledge about periodontitis, potentially attributed to the media campaign and visits to a specialist clinic in periodontology. Despite reporting mouth-related issues, patients rated their self-perceived oral health as relatively good. Prioritizing healthy teeth and improved well-being emerged as significant concerns for the patients. A positive relationship and trust in caregivers appeared to correlate with patient satisfaction.
**S**	Olofsson 2012 *Violence through the life cycle: A public health problem* Social Medicine and Public Health Science, Division of Community Medicine, Department of Medical and Health Sciences, Linköping University, Sweden	The general aim of this thesis is to describe the relation between exposure to violence or threats of violence and ill health at different ages and in different time periods of life.	Quantitative	Survey	Counities of Västernorrland, Jämtland, Västerbotten and Norrbotten, Sweden	People aged 0–84 years. Paper 1: Women aged 18–64 years and children 0–18 years Paper 2: Men and women aged 18–25 years. Paper 3: Men and women aged 65–84 years.	Exposure to violence, regardless of gender, emerges as a critical risk factor for poor health outcomes, warranting increased focus in public health initiatives. Robust associations between violence and diverse health outcomes were consistently observed across various life stages.
**F**	Pyae 2020 *The Use of digital games to enhance the physical exercise activity of the elderly: A case of Finland* Turku Centre for Computer Science, University of Turku, Department of Future Technologies, Turko, Finland	This study aimed at investigating digital games to specifically assess their applications for older Finns’ physical activities, focusing on the quality of users’ experiences, and their reported ease of use and perceived usefulness.	Mixed methods	Observation, interviews, and questionnaires	Finland and Japan	21 elderly Finns (14 women and seven men, average age = 76 years) We also conducted a comparative test in Japan with 24 elderly Japanese participants (12 women and 12 men, average age = 72 years) to further understand non-Finnish elderly users’ experiences	The use of digital games to deliver physical exercises to older adults. The findings not only align with existing literature but also offer new insights for future studies in designing and developing digital game-based exercises for older adults.
**S**	Sjöblom 2020 *Health promotion through the lifespan - the phenomenon of the inner child reflected in childhood events - experienced by children, adults and older persons* Division of Health and Rehabilitation, Department of Health Sciencesm Luleå University of Technology, Sweden	Aim of this thesis was to describe and gain more knowledge about the phenomenon of the inner child reflected in human beings’ experiences of childhood in connection to health and well-being in the present and through the life course.	Qualitative	Open-ended interviews drawing to support their narrations	Study 1: Medium-sized city in the central part of Sweden. Study 2: Primary school Study 3: Organization for senior citizens	Study 1: People aged 9–12 years. Study 2: People aged 22–68 years. Study 3: People aged 70–91 years. Study 4: People aged 9–91 years	Understanding the inner child’s role in health and well-being can inform health promotion strategies aimed at enhancing health literacy, underscoring the need for continued research in this area.
**N**	Sundsli 2015 *Engagement, knowledge, and autonomy – facing a new generation older urban living people: Studies on self-care and health* Norwegian University of Science and Technology, Faculty of Social Sciences and Technology Management, Department of Social Work and Health Science, Trondheim, Norway	To explore the phenomenon of self-care among older, urban home dwelling people to enhance health and well-being and be able to inform and improve policy and practice	Qualitative and quantitative	Questionnaire (examining undernutrition, self-care ability, agency, sense of coherence, mental health, background variables and health related questions), individual interviews and RCT to evaluate the effect of a telephone-based self-care intervention.	Living at home (with or without home care)	Study I: 1044 older adults living in urban areas aged 65+ years. Study II: 10 physically active older adults aged 65–82 years. Study III: Nine single living older adults aged 70–82 years and in good health. Study IV: 15 older adults aged 75–93 years.	Promoting good mental health, life satisfaction, physical activity, and preventing undernutrition are key factors in fostering self-care and overall health among older adults living in urban homes. Telephone-based interventions have the potential to enhance mental health, a crucial component of self-care. There’s a pressing need for society to recognize and involve older adults more comprehensively.
**N**	Tøien 2019 *An exploration of how long-term preventive home visits affect older persons’ health and possibility for a good life in their own homes. Users’ and service-providers’ perspectives* Institute of Health and Society, Department of Nursing Science, University of Oslo, Oslo, Norway	To explore how long-term preventive home visits affect older persons’ health and possibility for a good life in their own homes. Users and service users’ perspectives	Mixed methods	Individual research interviews and survey	Norwegian preventive home visit service in one municipality	Interviews with 10 older adults aged 81–91 years and 10 experienced nurses who had offered annual home visits after six or more years. A Survey of 161 service users (73 women and 88 men)	Preventive home visits offered personalized support, enhancing older individuals’ sense of security, self-esteem, and capacity to navigate daily life independently in their own homes. A comprehensive model was devised to inform the implementation, enhancement, and assessment of preventive home visit services.
**D**	Vorup 2017 *Health and physiological adaptations of small-sided team sport games in untrained older adults aged 65-93 years* University of Copenhagen, Department of Nutrition, Exercise and Sports, Denmark	The overall aims of the present thesis were to examine physiological adaptations important for health after a period of small-sided ball game training and protein ingestion in untrained elderly (+65 years), and to investigate if small-sided ball game training is feasible and can be considered as a health promoting activity for untrained elderly.	Quantitative	Three 12-week randomized controlled training studies examining the effect of small-sided ball game training on physical function, body composition, aerobic capacity, blood lipids and insulin resistance as well as markers of systemic inflammation in untrained elderly were conducted.	Danish municipality and elderly citizen´s homes	Older adults (men/women, aged 65–93 years)	Participating in small-sided ball games for 16–24 minutes twice a week resulted in significant physiological adaptations beneficial for health and physical function among untrained older adults aged 65–93 years. These adaptations have the potential to mitigate the risk of age-associated chronic diseases. Therefore, incorporating small-sided ball game training is recommended as a health-promoting activity for this demographic.
**S**	Wennerberg 2017 *Unravelling the duality of Caregivinghood* *How informal caregivers describe their situation when salutogenically approached* Institute of Health and Care Sciences Sahlgrenska Academy University of Gothenburg	The overall aim of the study in this thesis was to derive congruent knowledge concerning what informal caregivers’ Specific and Generalized Resistance Resources, SRRs/GRRs and Deficits, SRDs/GRDs may consist of, and to suggest how such knowledge may be used to promote their health.	Qualitative	Face to face interviews	A Swedish municipality	Informal caregivers aged 50–87 (mean age 71 years) (*n* = 32) Care recipients aged 63–97 years (mean age 77) (*n* = 32)	The concept of Caregiving hood is viewed as a continuum, akin to the salutogenic health ease/dis-ease continuum. Access to resources or experiencing deficits influences a person’s position on this continuum. According to salutogenic theory, specific resource reserves (SRRs) and specific resource deficits (SRDs) shape the strength of a person’s sense of coherence (SOC). A strong SOC is linked to positive health outcomes, highlighting the importance of understanding these resources and deficits. Therefore, health promotion should be both individualized and generalized, focusing on preserving SRRs and generalized resistance resources (GRRs), eliminating SRDs and generalized resistance deficits (GRDs), and providing GRRs when they are lacking.
**S**	Wiklund-Axelsson 2015 *Prerequisites for sustainable life style changes among older persons with obesity and for ICT support* Division of Health and Rehabilitation, Department of Health Sciences, Luleå University of Technology, Luleå, Sweden	The overall aim of this thesis was to explore the prerequisites for sustainable lifestyle changes among older persons with obesity and how this could be supported by ICT.	Qualitative and quantitative	Individual interviews, focus groups, and a randomly selected population survey	Participants´ home in northern Sweden	Older persons with obesity	Lifestyle changes prompted by obesity in later life demand vigilance, endurance, and effort. The potential of ICT to aid these changes, particularly from a long-term psychosocial standpoint, is promising. Findings underscore the necessity of shifting focus from weight to holistic health, highlighting opportunities and the significance of psychosocial support and enjoyable physical activity for lasting transformation.
**S**	Zingmark 2015 *Occupation-focused and occupation-based interventions for community-dwelling older people: Intervention effects in relation to facets of occupational engagement and cost effectiveness* Department of Community Medicine and Rehabilitation, Occupational Therapy, Umeå University, Sweden	The aim of this thesis was to evaluate the effects and cost effectiveness of occupation-focused and occupation-based interventions for two groups of community-dwelling older people, independent-living, community-dwelling older people and older people with bathing disabilities.	Quantitative	Exploratory randomized controlled trial, municipality-based trial, and decision-analytical model	Home of the older adults	Independent-living, community-dwelling older adults and older adults with bathing disabilities	Occupation-focused and occupation-based interventions can help older adults living independently in the community reduce their decline in occupational engagement and improve their self-rated health. Additionally, these interventions have the potential to be cost-effective. The research also shows that such interventions are effective for older adults with bathing disabilities, promoting independence from home help. Finally, interventions that increased the likelihood of being independent of home help for bathing positively impacted long-term QALYs and reduced societal costs, making them highly cost-effective.

#### The main aims in the included theses

The main aims of the included theses were examined to identify their focus (Table 1). In most of the theses, the contribution of knowledge was directly related to strengthening health promotion among older adults. However, there was also an interest in promoting an understanding of different phenomena related to aging. Most of the theses aimed to explore and/or evaluate health promotion interventions or programs, and their effects on various outcomes among older adults. These included both newly developed interventions as well as existing or adapted health promotion programs. The focus of the remaining theses was to gain a deeper understanding of factors associated with healthy aging, including different phenomena related to health and well-being among older adults, personal health resources, ethical questions, and the influence of the physical or social environment – only one thesis aimed to study the effects of health policies. The focus of the included theses is described in more detail below.

Based on their focus, the theses were divided into two main categories:

- Health promotion interventions (15 theses)
  - Comprehensive interventions (9 theses)
  - Healthy lifestyle interventions (6 theses)
- Determinants of healthy aging (13 theses)
  - Personal resources and different phenomena related to healthy aging (7 theses)
  - Influence of the environment on health (3 theses)
  - Ethics and policies (3 theses)

##### Theses focusing on health promotion interventions

According to the main aims, different health promotion programs or interventions were explicitly studied in 15 theses. The interventions/programs could be characterized as Comprehensive interventions (*n* = 9) and Healthy lifestyle interventions (*n* = 6).

*Comprehensive interventions* included a range of (multicomponent) health promotion efforts. Three theses focused on interventions related to frailty and/or the end of life (Gustafsson, 2012; Behm, 2014; Kleijberg, 2021), with outcomes including health, frailty, Activities of Daily Living, end-of-life issues, and/or intergenerational interactions. Two theses studied preventive home visits (Fjell, 2021; Tøien, 2019) and occupation-focused and-based interventions (Zingmark, 2015; Larsson, 2016). The outcomes encompassed health, the possibility of a good life in one’s own home, occupational engagement, and/or participation in society. Furthermore, two theses focused on interventions for older adults aging in migration (Barenfeld, 2016; Arola, 2018). Arola (2018) examined outcomes such as sense of coherence, life satisfaction, and engagement in activities, whereas Barenfeld (2016) was interested in how to support the development of a health promotion program for older adults aging in migration. In addition to Barenfeld (2016), three other theses studied the intervention process itself: Kleijberg (2021) investigated the processes of developing the intervention (DöBra) for the end of life, and Fjell (2021) examined the content of the preventive home visits. Within an occupational science framework, Zingmark (2015) evaluated the cost-effectiveness of occupation-focused interventions.

*Healthy lifestyle interventions* focused on oral health interventions (Mårtensson, 2012; Komulainen, 2013), physical activity interventions (Vorup, 2017; Pyae, 2020), prevention of undernutrition in-home care (Aure, 2021), and sustainable lifestyle changes among older adults with obesity (Wiklund-Axelsson, 2015).

We also found that information and communication technology (ICT) was used in health promotion research among older adults. ICT was part of the health promotion intervention in four of the 15 theses (Wiklund-Axelsson, 2015; Larsson, 2016; Pyae, 2020; Aure, 2021). Three of these theses focused on healthy lifestyles: there was interest in how lifestyle changes can be supported by ICT (Wiklund-Axelsson, 2015), how digital games can be used as a physical activity intervention (Pyae, 2020), and the feasibility of introducing an app to prevent undernutrition in home care (Aure, 2021). However, one of the theses using ICT focused on how support from a social internet-based occupational therapy intervention can contribute to social participation and healthy aging (Larsson, 2016).

##### Theses focusing on determinants of healthy aging

The rest of the 28 included theses (13 theses) focused on personal resources and different phenomena related to healthy aging (7 theses), the influence of the environment on health (3 theses), and ethical questions and policies (3 theses).

Seven theses investigated *personal resources and various phenomena related to healthy aging*, such as individual life experiences and health resources, and their relation to health, well-being, and health promotion (Mahler, 2012; Björklund, 2015; Grundberg, 2015; Sundsli, 2015; Boman, 2016; Wennerberg, 2017; Sjöblom, 2020). There was interest in the phenomenon of the ‘inner child’ (Sjöblom, 2020), inner strength as a health resource among older women (Boman, 2016), and informal caregivers’ health resources (Wennerberg, 2017). Moreover, there was a focus on gaining a deeper understanding of mental health and its promotion among older adults (Grundberg, 2015). The phenomenon of self-care (Sundsli, 2015) and the contextual phenomenon and experience of falling (Mahler, 2012) were studied, as well as the significance of temporal patterns of daily occupations and personal projects for older adults’ health and well-being (Björklund, 2015).

*The influence of the environment on health* was the focus in three theses (Forsman, 2012; Olofsson, 2012; Laatikainen, 2019). Laatikainen (2019) examined how multiple levels of factors, i.e., different physical environments together with individual factors, can support healthy and active aging, whereas Olofsson (2012) studied the relation between exposure to violence or threats of violence and ill health. Forsman (2012) investigated the promotion of mental health and well-being among older adults, with an interest in the influence of the social environment in terms of social capital and psychosocial interventions.

Three theses elucidated *ethical questions and policies*. Lood (2015) explored ethical and empirical points of departure for health promotion in relation to aging persons who have experienced international migration, whereas Ljungquist (2018) aimed to deepen the understanding of ethics and describe the actions and habits that are important in the ‘acts of caring’. Clotworthy (2017) was the only doctoral student to study policies and investigated how political goals and individualized health policies influence the provision of in-home health services for older adults.

#### Theoretical perspectives

The included theses were examined for their main theoretical perspective (Table 2). This provided information on (a) whether the thesis had a health promotion approach, (b) the theoretical foundations for the chosen perspective, and (c) if the chosen theory was compatible with a health promotion ideology. Of the 28 included theses, three used a comprehensive and very broad understanding of health promotion, referring to it as any activity that improves health status (Laatikainen, 2019; Tøien, 2019; Fjell, 2021). Fourteen theses had a health promotion approach based on the Ottawa Charter (Forsman, 2012; Gustafsson, 2012; Mårtensson, 2012; Mahler, 2012; Komulainen, 2013; Behm, 2014; Björklund, 2015; Lood, 2015; Zingmark, 2015; Barenfeld, 2016; Wennerberg, 2017; Sjöblom, 2020; Aure, 2021; Kleijberg, 2021). Eight theses did not define health promotion (Olofsson, 2012; Wiklund-Axelsson, 2015; Boman, 2016; Larsson, 2016; Clotworthy, 2017; Vorup, 2017; Ljungquist, 2018; Pyae, 2020). Three theses (Grundberg, 2015; Sundsli, 2015; Arola, 2018) used other definitions for health promotion (see Table 2). Several theses had supplementary theoretical perspectives. A salutogenic approach, where health is viewed as a resource and ongoing process, was applied in four theses (Mahler, 2012; Björklund, 2015; Boman, 2016; Wennerberg, 2017; Arola, 2018).

**Table 2.** Theory, definition of health promotion, and selected guiding principles of health promotion recommended by the World Health Organization European working group on health promotion evaluation (Rootman, 2001). (D = Denmark, F = Finland, N = Norway, S = Sweden)

	Author, year	Theory	Definition/understanding of health promotion	Empowering (empow*, enabl* mentioned x times (t) in the thesis)
**S**	Arola 2018	A salutogenic and occupational approach to describe health and health resources-> able to develop health promotion interventions with a focus on a person’s capabilities-> Person-centered approach in health promotion. Relevant health promotion references are provided.	Health promotion is about empowering people participating in health promotion interventions, with the aim to maintain or improve their health and wellbeing (Korp, 2016).	Empow*= 12 t. in total: Found in background, rationale, methodology, and discussion. Enabl*= 23 t. in total: Found in title, background, discussion, conclusion, and practical implications.
**N**	Aure 2021	The Socio-ecological model (Booth *et al*., 2001; Sallis and Neville, 2015)	‘Health promotion aims at supporting health experiences, well-being, and quality of life’, Aglen *et al*., 2018. The WHO defines health promotion as ‘the process of enabling people to increase control over, and to improve their health’ (WHO, 1986).	Empow*= 8 t. in total: Found in background, discussion, and paper 1 and 3. Enabl*= 24 t. in total: Found in abstract, introduction, background and discussion.
**S**	Barenfeld 2016	Capability approach (Sen, 1993) ‘Knowledge translation’ - Knowledge To Action (KTA) framework (Graham *et al*., 2006 ). A definition of health promotion is provided based on the Ottawa Charter.	Health promotion is a strategy for improving public health. It has been defined as a process to enable individuals and communities to increase control over or improve their health, Wilcock, 1998 In this thesis, health promotion refers to the administration of a health-promotion program aimed to enable older adults to manage everyday life.	Empow*= 2 t. in total: Found in discussion. Enabl*= 19 t. in total: Found in background, rationale, discussion and implications for practice and research.
**S**	Behm 2014	Health belief model (Janz and Becker, 1984) Trans-theoretical model of change (Prochaska and DiClemente, 1983). The author provides different understandings of health promotion and disease prevention. The author uses health-promotion and disease prevention side by side throughout the thesis.	Health promotion has been defined as ‘the process of enabling people to increase control over their health and its determinants, and thereby improve their health’ (WHO, 1986).	Empow*= 12 t. in total: Found inbackground, methodology, discussion, and conclusion. Enabl*= 6 t. in total: Found in background, methodology, results, and discussion.
**S**	Björklund 2015	Salutogenic perspective sense of coherence (Antonovsky, 1996)	‘…health promotion will enable people to increase control of improving health as an on-going process of identifying aspirations, realizing goals, and satisfying needs in life, within their environment. When health is viewed as a resource and ongoing process, the responsibilities of health promotion go beyond the health sector (WHO, 1986)’. ‘…health promotion influenced by active aging calls for older people to be in control of their lives, to identify their aspirations, to realize goals and to satisfy their needs in life by being active participants in society, despite functional incapacities’.	Empow*= 0 t. Enabl*= 1 t. Found in methods.
**S**	Boman 2016	Salutogenic perspective ‘Theoretical model where inner strength comprises four interrelated and interacting dimensions: connectedness, creativity, flexibility, and firmness, and being rated by the Inner Strength Scale (ISS) (Lundman *et al*., 2011)’ ‘The theoretical framework of health-related quality of life (HRQoL) (Bowling, 2001)’	No definition of health promotion.	Empow*= 0 t. Enabl*= 0 t.
**D**	Clotworthy 2017	A phenomenological perspective, wherein the body is understood as part of a historical person who engages with, lives in, and experience their world in a particular way. The analysis of the empirical material is largely influenced by the work of philosopher Michel Foucault and political theorist Hannah Arendt.	No definition of health promotion.	Empow*= 84 t. in total: Found in title, abstract, table of contents, introduction, methods, results/discussion, conclusion, and reference list. Enabl*= 30 t. in total: Found in abstract, introduction, method, results/discussion, and conclusion
**S**	Fjell 2021	Health: ‘According to the traditional biomedical understanding, health is the absence of illness and functional impairment (Huber *et al.*, 2011; Jans-Beken *et al*., 2019)’ Healthy aging (WHO, 2015)	‘A comprehensive and holistic understanding of health promotion as any activities that improve health status (Tones and Green, 2004)’	Empow*= 0 t. Enabl*= 1 t.: Found in abstract.
**S**	Forsman 2012	Social capital as a theoretical concept Bourdieu’s theoretical framework of social capital (Bourdieu, 1986) Putnam’s perspective, social capital (Putnam, 1993)	‘Health promotion was defined at the first international conference on health promotion in Ottawa in 1986 (WHO, 1986) as a process of enabling people to improve and increase control over their health status.’ Mental health promotion: ‘The overall objective of mental health promotion is to strengthen and maintain the environmental, social, and individual factors that determine mental health, reaching the target group on macro (societal), meso (community) and micro (individual) levels of society (Lahtinen *et al*., 1999).’	Empow*= 2 t.: Found in abstract and conclusion. Enabl*= 23 t.: Found in abstract, introduction, overview of study design and methods, ethical considerations, and discussion.
**S**	Grundberg 2015	No theory explained.	Mental health promotion: ‘mental health promotion involves actions to create living conditions and environments that support mental health and allow people to adopt and maintain healthy lifestyles’ (WHO, 2014). ‘Health promotion is further described as an umbrella term among professionals, where disease prevention and health-promoting activities are included as well as a concept with a salutogenic approach.’ ‘In this thesis, mental health promotion is related to the old persons’ subjective view of what may influence mental health as well as DNs (District nurses) and HCAs (Home Care Assistants) perspective of what may promote mental health among the population in focus.’	Empow*= 0 t. Enabl*= 0 t.
**S**	Gustafsson 2012	International Classification of Functioning Disability and Health (ICF) as a structural framework	‘…health promotion is the process of enabling people to increase control over, and to improve their health, while disease prevention covers measures not only to prevent the occurrence of disease, such as risk factor reduction, but also to arrest its progress and reduce its consequences once established (WHO, 2009). Disease prevention, as defined by WHO, partially overlaps the concept of health promotion for persons with chronic diseases, enhancing people’s capacity to cope with chronic conditions (Kickbusch, 1992). Consequently, the definition of health promotion used in this thesis covers both health-promoting and disease-preventive (HPDP) initiatives, which typically contain multi-component or complex interventions (several interacting components)’	Empow*= 0 t. Enabl*= 1 t. Found in future research.
**S**	Kleijberg 2021	Modified play theory	‘Health promotion is defined based on the Ottawa Charter: the process of enabling people to increase control over, and improve, their health (WHO, 1986)’.	Empow*= 9 t. in total: Found in introduction/aim, situating this thesis methodologically, and discussion. Enabl*= 7 t. in total: Found in background and summary of findings
**F**	Komulainen 2013	No theory explained	Definition of key terms in the thesis: Oral health promotion: ‘Health promotion is a process of enabling people to increase control over and improve their health through education, prevention, and health protection’.	Empow*= 1 t.: Found in discussion. Enabl*= 4 t.: Found in review of literature
**F**	Laatikainen 2019	An ecological model of Physical Activity (PA) (Sallis and Owen, 2015): Transactional worldview (Altman and Rogoff, 1987), Ecological analysis of health behavior and health-promotive environments (Stokols, 1992, 1996) and understanding the human-environment interaction (Gibson, 1986).	Health, wellbeing, and health promotion defined based on the definition by WHO (2004). Health promotion definition in the thesis: … ‘any organizational, political, practical or societal aim to improve or protect health’.	Empow*= 0 t. Enabl* = 6 t. in total: Found in introduction, methods, results/discussion, and conclusion.
**S**	Larsson 2016	This thesis is grounded in the belief that participation in meaningful activities can contribute to the individuals’ health through interactions with others and the skills and experiences that are obtained (Law, 2002). The following three main perspectives are frequently used to explain social and activity transitions during aging: the activity theory, the continuity theory, and the disengagement theory (Utz *et al*., 2002). OT theory states that successful interventions should be based on clients’ goals and individual adaptations of the intervention processes (Kielhofner, 2008). The theoretical guidance of this intervention is the individual and client-centered approach described in the Occupational Therapy Intervention Process Model (OTIPM) (Fisher, 2009)	No definition of health promotion.	Empow*= 1: Found in discussion. Enabl*= 1: Found in introduction
**F**	Ljungquist 2018	The design of the study is based on and from a human-scientific perspective and founded on a hermeneutic tradition based on Gadamer’s philosophy. The caritative care theory. The data has been analyzed and interpreted by means of qualitative content analysis.	No definition of health promotion.	Empow*= 2 t. in total: Found in the comprehensive summary of the thesis, written in English. The thesis was written in Swedish. Enabl*= 0 t.
**S**	Lood 2015	Wilcock theory of human need for occupation (Wilcock, 1993)	‘Health promotion has been defined as a process to enable individual persons, and communities to increase control over their health (WHO, 1986).’	Empow*= 2 t. Found in introduction. Enabl*= 4 t. Found in introduction and discussion
**S**	Mahler 2012	Interpretative phenomenology as philosophical and analytical method was used. The thesis has several theoretical perspectives, e.g. Critical Gerontology (Holstein & Minkler 2007) and Life Course research (Settersten, 2003).	The Public Health understanding is embedded in the views of the Ottawa Charter (WHO, 1986). Health Promotion includes participant-orientation and dialog-based processes with a view to promoting ownership and competence to act (Jensen, 2009; WHO, 1998). Health promotion has a salutogenetic perspective (Antonovsky, 1987; Eriksson, 2007)	Thesis in Danish. Danish search terms for empow* and enabl* used. The Danish terms ‘i stand til’ found in introduction and ‘mestre’ found in methods.
**S**	Mårtensson 2012	No theory explained.	‘Health promotion is a strategy for improving health and well-being in a population. WHO (1986) defined health promotion as a process of enabling people to increase control over and improve their health’. Health promotion can be seen as a social and political process to make it possible for people to control factors that increase their health (Nutbeam, 1998)	Empow*= 1 t. Found in introduction. Enabl*= 3 t. Found in introduction.
**S**	Olofsson 2012	Violence theoretical framework (Krug *et al*., 2002; Brundtland, 2002)	No definition of health promotion.	Empow*= 0 t. Enabl*= 0 t.
**F**	Pyae 2020	The philosophy of the pragmatic worldview is applied.	No definition of health promotion.	Empow*= 1 t.: Found in methodology. Enabl*= 7 t.: Found in paper 2 and 7, and introduction, and background
**S**	Sjöblom 2020	Hermeneutic phenomenological	Health promotion focuses on achieving equity in health. According to the Ottawa Charter (WHO, 1986), health is created by caring for ourselves and others, being able to make decisions and have control over one’s life circumstances and ensuring that the society one lives in creates conditions that allow for the attainment of health by all its members (WHO, 1986).	Empow*= 0 t. Enabl*= 0 t.
**N**	Sundsli 2015	Self-care Theory (Orem, 2001) and Salutogenesis (Antonovsky, 1987)	Self-care as a scientific health approach in health promotion: ‘Health promotion as a scientific discipline finds its roots in interdisciplinary research that accomplish the formulation of the WHO’s principle on health, that is more than the absence of disease or infirmity’ (WHO, 2007). ‘Self-care (Söderhamn, 1998) and salutogenesis (Antonovsky, 1979) both point in the direction of health rather than disease, and therefore contribute to the core in the health promotion rationale created in the Ottawa Charter of 1986 (WHO, 1986).’	Empow*= 0 t. Enabl*= 17 times in total: Found in background, discussion and conclusion.
**N**	Tøien 2019	Different theories/models are presented but it’s emphasized that they do not form a coherent framework. However, the thesis is based on a comprehensive understanding of health promotion.	In this study, we hold a comprehensive understanding of health promotion as ‘any activity that improves health status’ (Green, 2015).	Empow*= 9 t. in total: Found in background and discussion. Enabl* = 11 t. in total: Found in background, results, and discussion.
**D**	Vorup 2017	No theory explained.	No definition of health promotion.	Empow*= 0 t. Enabl*= 0 t.
**S**	Wennerberg 2017	‘Informal caregiving research, health promotion and salutogenesis’	Health promotion is based on the Ottawa Charter for health promotion (WHO, 1986) and on salutogenic research based on Antonovsky´s Sense of Coherence (Eriksson, 2007)	Empow*= 0 t. Enabl*= 7 t.: Found in introduction, discussion, and reference list.
**S**	Wiklund-Axelsson 2015	Self – Determination Theory, Self-efficacy, COM-B system, and Technology acceptance models	No definition of health promotion.	Empow*= 15 t.: Found in content, introduction, discussion, conclusion, and reference list. Enabl*= 1 t.: Found in discussion and reference list.
**S**	Zingmark 2015	Demographic development, successful aging, theoretical perspectives on health, occupational perspectives on health, and occupational transitions.	Health promotion refers to the process of enabling people to increase control over and to improve, their health (WHO, 1986).	Empow*= 0 t. Enabl*= 0 t.

##### Theories compatible with a health promotion ideology

Some of the included theses had a health promotion focus throughout the comprehensive summary of the thesis and referred to central health promotion theorists. Mårtensson (2012) and Sjöblom (2020) referred to empowerment ideologies and central health promotion theorists such as Kickbusch *et al*. (2005) and Nutbeam (2008). Several authors used supplementary theoretical perspectives, which were either compatible with health promotion principles (Forsman, 2012; Komulainen, 2013; Grundberg, 2015; Sundsli, 2015; Boman, 2016; Barenfeld, 2016; Arola, 2018) or used a theoretical model to provide a descriptive framework (Gustafsson, 2012; Laatikainen, 2019) that was not in conflict with health promotion ideologies.

Self-care, as an approach to health promotion, provided the theoretical background in Sundsli’s (2015) research. This approach was based on the WHO’s definition of health as more than the absence of disease (WHO, 2007). The capability approach provided the theoretical background in Arola’s (2018) thesis, which related to empowering individuals to participate in health promotion interventions and to maintain or improve their health and well-being, with Korp (2016) used as a reference. Barenfeld (2016) also referred to the capability approach and regarded health promotion as a strategy for improving public health.

Laatikainen (2019) focused on an ecological model of health (Sallis *et al*., 2006), whereas Boman (2016) referred to a theoretical model based on inner strength within a framework of health-related quality of life. Forsman (2012) referred to social capital as a theoretical concept and applied a definition of health promotion based on the Ottawa Charter (WHO, 1986) and a definition of mental health promotion created by Lahtinen *et al*. (1999). Mental health promotion was also the focus of Grundberg’s (2015) thesis and was defined as ‘actions to create living conditions and environments that support mental health and allow people to adopt and maintain healthy lifestyles’ (197). Gustafsson (2012) used the International Classification of Functioning, Disability, and Health (ICF) as a structural framework. Kleijberg (2021) used modified play theory. Zingmark (2015) had a health promotion focus throughout and referred to health, occupational perspectives on health, and occupational transitions. Komulainen (2013) defined oral health promotion as ‘enabling people to increase control over and improve their health through education, prevention, and health protection’.

##### The concept of health promotion is poorly defined

Several of the included theses did not define the concept of health promotion (Olofsson, 2012; Wiklund-Axelsson, 2015; Boman, 2016; Larsson, 2016; Clotworthy, 2017; Vorup, 2017; Ljungquist, 2018; Pyae, 2020). Wiklund-Axelsson (2015) referred to self-determination theory, self-efficacy, the COM-B system, and the Technology Acceptance Model. Clotworthy (2017) was largely influenced by the works of philosopher Michel Foucault and political theorist Hannah Arendt. Vorup (2017) did not provide a theoretical framework or a definition of health promotion. Others referred briefly to a definition but did not use the concept as a theoretical framework. Larsson (2016) used several theoretical approaches, and although health promotion was not defined, there was a focus on healthy aging. Ljungquist (2018) did not define health promotion but applied caritative care theory (Eriksson, 1995), which is based on health promotion ideologies within a caring science tradition. Pyae (2020) provided a pragmatic worldview as a theoretical perspective and referred to different reports written by the WHO, emphasizing the importance of participation and the promotion of a healthy and independent lifestyle. The pragmatist worldview could be seen as compatible with health promotion principles.

##### The use of the concepts ‘participation’ and ‘empowerment’

Participation is an important guiding principle in health promotion. However, it appears that the older adults involved in the various research projects did not participate in designing the interventions or provide input on what they considered relevant research focuses. Only one thesis (Kleijberg, 2021) among the 28 noted their participation. Furthermore, only one thesis explicitly related its results to empowerment (Clotworthy, 2017) – another key principle in health promotion. In more than half of the theses, empowerment was mentioned, but mainly in the introduction and/or discussion sections. A few theses drew conclusions related to empowerment (see Table 2).

#### Methods used in the theses

The research methods used in the theses were a combination of qualitative and quantitative approaches (*n* = 12), followed by qualitative methods alone (*n* = 7), quantitative methods alone (*n* = 5), and mixed methods (*n* = 4).

##### Context/Population

Most participants were healthy, home-dwelling individuals older than 65 years, both men and women, living either in urban or rural areas. Only one focus group interview took place in a nursing home, in Finland (Forsman, 2012). The sample sizes in these theses ranged from around ten participants (Mahler, 2012; Grundberg, 2015; Sjöblom, 2020) to thousands (Olofsson, 2012).

The studies were conducted in a Nordic context with predominantly Nordic participants. However, three Swedish theses included migrants from the Western Balkans (Barenfeld, 2016; Arola, 2018) and migrants from Bosnia, Herzegovina, Croatia, Montenegro, and Serbia (Lood, 2015), in addition to migrants from Finland.

#### Key conclusions drawn in the theses

The main conclusions sections were examined in the theses (Table 1). Primarily positive conclusions were drawn and related mainly to various health promotion interventions.

Theses on health promotion interventions such as preventive home visits, senior meetings, digital interventions, and interventions focusing on oral health, occupation, and falls concluded that the interventions could postpone frailty and declining health (Behm, 2014), and occupational engagement (Zingmark, 2015), delay deterioration and reduce dependency (Gustafsson, 2012). Furthermore, positive conclusions were related to improved self-rated health (Zingmark, 2015), oral health status (Komulainen, 2013), mental health, self-care (Sundsli, 2015), feeling safer, increased self-worth, ability to manage everyday life (Behm, 2014), and sustainable lifestyle changes (Wiklund-Axelsson, 2015). Other conclusions were the importance of promoting physical activity (Pyae, 2020), prevention of undernutrition (Aure, 2021), motivation of healthier behavior (Behm, 2014), identifying capabilities (Arola, 2018), support of social activities and contacts (Larsson, 2016), and cost-effectiveness (Zingmark, 2015).

Aspects that are important to reflect upon in health-promotive interventions to promote health and well-being were also emphasized in the conclusions. It seemed important to tailor interventions to suit the personal needs of older adults (Tøien, 2019) and address older adults’ perspectives (Forsman, 2012; Grundberg, 2015; Larsson, 2016). Examples were given to involve older adults in planning initiatives and creating a health-promoting dialog based on the person’s wishes and needs (Grundberg, 2015). Moreover, important aspects were to bring the intervention home to the person (Komulainen, 2013), have a good attitude as personnel (Ljungquist, 2018) and create a good relationship (Mårtensson, 2012). Furthermore, one thesis concluded that the interplay between personal choices and normative power was important in promoting health (Lood, 2015). Other aspects important in health promotion were identifying risks (falls, malnutrition, polypharmacy, and cognitive impairment), self-rated health, social networks, activity (Fjell, 2021), physical training (Vorup, 2017), mapping occupations during 24-hour sequences (Björklund, 2015), rating inner strength (Boman, 2016), contextualizing falling accidents (Mahler, 2012) and assessing the physical environment (Laatikainen, 2019).

## Discussion

This study explored how research about older adults, under the label ‘health promotion’, was undertaken in doctoral theses within a Nordic context. The focus was on the theoretical framework, participation, and empowerment, two main guiding principles for health promotion. We found 28 theses focusing on health promotion among older adults from the years 2011 to 2021. Most of the theses involved participants living in their own homes and utilized both qualitative and quantitative research methodologies. The major focus in all theses was on health promotion interventions for older adults or on the determinants of healthy aging, ranging from personal resources to ethics and policies. The findings reveal that health promotion is predominantly defined in line with the Ottawa Charter (WHO, 1986). However, only one of the theses involved older adults in the research process (Kleijberg, 2021), and empowerment was mentioned in the results section of only one thesis (Clotworthy, 2017).

The findings have the potential to shape future research and health-promoting interventions for older adults in the Nordic countries. Researchers may find these insights particularly valuable, given the lack of clear definitions of health promotion in many theses and the inconsistent application of supporting theories. Key concepts like empowerment and participation were seldom implemented, indicating a gap between theoretical frameworks and practice. Only one doctoral thesis actively engaged older adults (in the research process) and only one linked outcome to empowerment. While most theses focused on community-dwelling older adults, only one addressed those in assisted living. Thus, this overview highlights the need for precise definitions, better integration of theory into practice, and broader research that includes populations beyond community-dwelling older adults. These findings could guide future researchers by emphasizing the need for clearer definitions and more consistent use of theories in theses. Additionally, the results identify research gaps, such as the predominant focus on health promotion interventions for community-dwelling older adults.

The main aims of doctoral theses may vary over time. The findings show that research in doctoral theses on health promotion and aging in Nordic countries encompasses both evaluations of interventions and more exploratory studies on various determinants of healthy aging. Less than half of the interventions focused on promoting healthy lifestyles, while more than half assessed comprehensive interventions, such as preventive home visits. In the included theses the lifestyle interventions in the Nordic doctoral theses addressed various aspects, such as physical activity, oral health, nutrition, and sustainable lifestyle changes based on psychosocial support and enjoyable physical activity. In contrast, the comprehensive interventions focused on diverse health-related outcomes, including general health, sense of coherence, life satisfaction, occupational engagement, participation in society, end-of-life issues, and frailty. According to earlier reviews on health promotion interventions among older adults (Duplaga *et al*., 2016), the most common target area was physical activity, followed by general health and quality of life. This trend was also observed in a subsequent review on health promotion and primary prevention by Chiu *et al*. (2020), excluding the ‘disease-oriented’ target area. Thus, it appears there has been less emphasis on physical activity in Nordic theses compared to earlier reviews on health promotion interventions for older adults during the same period. However, the present results provide a snapshot of the topics addressed in these theses during the period 2011–2021. Since then, additional theses have been produced in the field (Nivestam, 2022; Löfvendahl, 2025; Dinse, 2026), highlighting the ongoing exploration of new research areas. Therefore, the results should not be considered comprehensive, but rather as an overview that may inform and stimulate future discussion and research development.

The results indicated that the interpretation of health promotion varies significantly among the authors of the 28 included theses, and their theoretical perspectives exhibit considerable diversity. Certain guiding principles of health promotion are present in all the theses reviewed, based on enabling people to increase control over and improve their health. Rootman *et al*. (2001) suggested, based on a review of various definitions of health promotion, that ‘empowering activities’ could be regarded as the primary criterion for determining whether an initiative should be termed health promotion. Empowerment is considered both an outcome in itself and a significant step toward other health-related outcomes (Wallerstein and Duran, 2006). However, in practice, health promotion often encompasses a range of activities aimed at improving or maintaining health (Rootman *et al*., 2001). The theoretical literature and research emphasize the importance of using a theory in the development of health promotion interventions to achieve efficient outcomes (Haugan and Eriksson, 2021). To improve the quality of health promotion research within the field of aging and health, researchers may need to be clearer about the definition, how theory is underpinned in their research, and how it is connected to related concepts.

The participation of older adults in the development of health-promotive interventions was highlighted as a crucial factor in some of the theses’ conclusions sections. By involving older adults in the design and planning stages, interventions can be more effectively tailored to meet their specific needs and preferences (Malengreaux *et al*., 2022). A participatory approach can enhance the relevance and acceptability of the intervention. However, a recent review (Cowdell *et al*., 2022) shows, in line with our results, that older adults seldom participate throughout the entire process. Usually, they are only involved in providing information to develop the intervention. It is worth reflecting on how older adults could be involved in different stages of the development process.

Being involved in the research process may be one way of empowering people. In all included theses, older adults were invited to participate, but only (primarily) as informants and respondents during interviews and questionnaires. The relatively recent emergence of involving laypeople in the research process may explain this finding (Dengsø *et al*., 2023). Several authorities in the Nordic countries now emphasize that patients and the public should be involved in the research process from formulating the research question to the final product (Aas *et al*., 2023), to ensure that research is tailored to the needs of practice (Pedersen *et al*., 2022). However, research shows that stakeholders are more frequently involved in the planning and conduct of the studies and less in disseminating and implementing research results (Aas *et al*., 2023). Additionally, there is a risk of overrepresentation from better socio-economic environments (Pii *et al*., 2019). It is important to also include a diversity of older adults, for example, persons who are ‘hard to reach’ (e.g., older persons from minority ethnic groups) (Liljas *et al*., 2017) in health promotion interventions. This inclusion matters because these groups generally face higher health risks than the overall population (Liljas *et al*., 2017). Despite a growing interest in involving older adults in research projects, researchers often find their participation challenging, which can lead to their exclusion (Haak *et al*., 2021).

Another notable finding is that doctoral research on older adults in a Nordic context, specifically under the label of health promotion, was predominantly conducted in Sweden. An earlier scoping review shows a dramatic increase in systematic reviews focusing on health promotion among older adults since 2009 (Duplaga *et al*., 2016). Conversely, research indicates a decline in the practice and discipline of health promotion (Woodall *et al*., 2018). Woodall and Freeman (2020) argue that health promotion is an ambiguous concept, which might explain its reduced utilization. Despite its ambiguity, the term is frequently used in Swedish policies (SOU, 2020: 19) and in a recently published thesis with a particular focus on older adults (Nivestam, 2022). A similar focus on promoting health and well-being among older adults has been highlighted in Denmark (Sundhedsstyrelsen, 2024), Finland (Act for Elderly Care and Services 980/2012), and Norway (Regjeringa.no 2023). Our results showed that in Denmark, Finland, and Norway, the label health promotion was less frequently used in theses related to aging and health compared to Sweden. The same pattern was observed in a study by Eriksson *et al*. (2020), which explored theses in the Nordic countries focusing on ‘health promotion’ in general and found that more than double the number of theses were from Sweden. There could be several reasons for this, such as the use of related concepts instead of health promotion, like empowerment and well-being. Additionally, Sweden is a larger country with more universities compared to other Nordic countries. The Nordic School of Public Health (Suominen, 2014) was located in Sweden from 1953 to 2014, which may have inspired more researchers from Sweden to continue working with the concept of health promotion.

## Strengths and limitations

Conducting a scoping review has both strengths and limitations (O’Brien *et al*., 2016). The scoping review provided a good overview of the topic, and the results include theses with different kinds of methodologies. The study is an important contribution to the research field. Ericsson *et al*. (2020) highlighted that there are a limited number of reviews of Nordic health promotion research, including doctoral theses performed in a Nordic context. The study serves as a reminder to researchers in the field of health promotion to provide a clear definition of the concept.

The limitation of the scoping review is that there is no quality assessment made on the theses included, however, all theses have been assessed during a doctoral defense. Moreover, the aim of a scoping review is not to critically evaluate and synthesize results but rather to provide an overview of the evidence (Munn *et al*., 2018).

There are three major limitations in the searches made. First, to improve the search strategy, synonyms for ‘health promotion’ could be used. However, this study aimed to focus on the label ‘health promotion’. Still, to further investigate the reasons for a limited number of theses found in Denmark, Finland, and Norway, a broader search strategy could be applied, including synonyms and related concepts. Second, there was no standardized database for theses in the Nordic countries but different databases in each country, with some databases having limited search functions. The search processes were adapted for each available database. No search is exhaustive, and there is always the possibility that not all relevant theses were included in the analysis. Third, the searches were conducted in 2022, which means that more recent doctoral theses have been published since then, not included in the present results. We argue that the research has novel findings that provide a valuable snapshot of relevant health promotion research for the period 2011–2021 and provides a starting point for future research reviews focusing on new time frames. To further enhance the search strategy and obtain a more comprehensive overview, it may be beneficial to include searches of reference lists and gray literature, as well as theses from Iceland. Iceland was not included in the presents study because there was no researcher from Iceland in the group, and conducting database searches for theses requires local knowledge.

## Conclusions

In conclusion, this study illuminates doctoral thesis research on health promotion, particularly focusing on aging and health, in the Nordic countries. The analysis of theoretical frameworks shows varying approaches, from adherence to established models like the Ottawa Charter for Health Promotion to broader interpretations lacking clear definitions. While some theses have strong theoretical foundations, others lack clarity, indicating a need for coherence in conceptual frameworks. Despite the recognized importance of participation and empowerment, older adults often play passive roles in research. Few theses explicitly address empowerment, suggesting a possible gap in engaging older adults meaningfully. Greater emphasis on active participation and empowerment is necessary to ensure interventions meet older adults’ needs. Moving forward, research should prioritize the active involvement of older adults and clearer conceptual definitions. By fostering meaningful engagement, future research can develop more effective interventions tailored to older adults’ needs, improving health promotion outcomes in the Nordic context. Overall, integrating participatory approaches and robust theoretical frameworks is crucial for advancing understanding and improving outcomes in promoting the health and well-being of older adults in the Nordic region.

## Data Availability

No new data were generated or analyzed in support of this research.

## References

[ref2] Aas SN , Distefano MB , Pettersen I , Gravrok B , Nordvoll LY , Bjaastad JF and Grimsgaard S (2023) Patient and public involvement in health research in Norway: A survey among researchers and patient organisations. Research Involvement and Engagement 9(1), 48. 10.1186/s40900-023-00458-x 37422661 PMC10329785

[ref1] Act for Elderly Care and Services (980/2012) (2012) Act on supporting the functional capacity of the older population and on social and health care services for older persons. No. 980/2012. Finland. Retrieved 10 October 2024 from https://www.finlex.fi/fi/laki/kaannokset/2012/en20120980_20120980.pdf.

[ref87] Aglen BS , Olufsen V and Espnes G-A (2018) Helsefremming og sykdomsforebygging er ikke to sider av samme sak. Sykepleien 106, Article e70809. 10.4220/Sykepleiens.2018.70809

[ref96] Altman I and Rogoff B (1987) World views in psychology: Trait, interactional, organ ismic, and transactional perspectives. Handbook of Environmental Psychology 1, 7–40.

[ref109] Antonovsky A (1979) Health, Stress and Coping. San Francisco, CA: Jossey-Bass Publisher.

[ref126] Antonovsky A (1987) Unraveling the Mystery of Health: How People Manage Stress and Stay Well. San Francisco: Jossey-Bass.

[ref90] Antonovsky A (1996) The salutogenic model as a theory to guide health promotion 1. Health Promotion International 11(1), 11–18.

[ref3] Arola A (2018) Capturing the experience of health among persons aging in a migration context health promotion interventions as means to enable health and occupations in daily life. [University of Gothenburg], Sweden. Retrieved 10 September 2023 from: https://gupea.ub.gu.se/bitstream/handle/2077/56883/gupea_2077_56883_4.pdf?sequence=4.

[ref4] Aure CF (2021) Technology-Mediated Patient Engagement in Nutrition Care Opportunities for Early Intervention to Support Health and Independence in Old Age. [University of Oslo], Norway. Available at https://www.duo.uio.no/handle/10852/86360.

[ref5] Barenfeld E (2016) How to support knowling and doing in promotion of health lessons learned from the promoting aging migrants’ capabilities program. [University of Gothenburg], Sweden. Available at https://gupea.ub.gu.se/bitstream/handle/2077/47404/gupea_2077_47404_1.pdf?sequence=1.

[ref6] Behm L (2014) It’s never Too Late. Health-Promotion and Disease Prevention for Very Old Persons. [University of Gothenburg], Sweden. Avilable at https://gupea.ub.gu.se/items/2e4fc20c-e305-462a-9f99-ce870f860e57.

[ref7] Björklund C (2015) Temporal Patterns of Daily Occupations and Personal Projects Relevant for Older Persons’ Subjective Health - a Health Promotive Perspective. Luleå [University of Technology], Sweden. Available at https://ltu.diva-portal.org/smash/get/diva2:990870/FULLTEXT01.pdf.

[ref8] Blix BH and Hamran T (2021) Brukermedvirkning og representasjon i helse-og omsorgsforskning. Tidsskrift for omsorgsforskning 7(3), 1–15. 10.18261/issn.2387-5984-2021-03-07

[ref9] Bloom DE , Chatterji S , Kowal P , Lloyd-Sherlock P , McKee M , Rechel B , Rosenberg L and Smith JP (2015) Macroeconomic implications of population ageing and selected policy responses. Lancet 385(9968), 649–657. 10.1016/s0140-6736(14)61464-1 25468167 PMC4469267

[ref10] Bodin E , Kumlin L and Tengqvist A (2020) Att åldras i Norden. Retrieved 10 October 2024 from https://nordicwelfare.org/wp-content/uploads/2020/11/FINAL_rapport-Att-aldras-i-Norden.pdf.

[ref11] Boman E (2016) *Inner strength as a health resource among older women*, [Umeå University], Sweden. Available at https://www.diva-portal.org/smash/get/diva2:1105141/FULLTEXT01.pdf.

[ref86] Booth SL , Sallis JF , Ritenbaugh C , Hill JO , Birch LL , Frank LD , Glanz K , Himmelgreen DA , Mudd M , Popkin BM , Rickard KA , St. Jeor S and Hays NP (2001) Environmental and societal factors affect food choice and physical activity: Rationale, influences, and leverage points. Nutrition Reviews 59(3), 21–36. 10.1111/j.1753-4887.2001.tb06983.x. 11330630

[ref118] Bourdieu P (1986) The forms of social capital. In Richardson, J. (Ed.), Handbook of Theory and Research for the Sociology of Education. New York: Greenwood, pp. 241–248.

[ref115] Bowling A (2001) Measuring Disease: A Review of Disease-Specific Quality of Life Measurement Scales, 2nd Edn. Buckingham: Open University Press.

[ref107] Brundtland GH (2002) Preface. In Dahlberg LL, Krug EG, Mercy JA, Zwi AB and Lozano R (Eds.), World Report On Violence And Health. Switzerland: World Health Organization.

[ref12] Chang AY , Skirbekk VF , Tyrovolas S , Kassebaum NJ and Dieleman JL (2019) Measuring population ageing: An analysis of the Global Burden of Disease Study 2017. Lancet Public Health 4(3), e159–e167. 10.1016/s2468-2667(19)30019-2 30851869 PMC6472541

[ref13] Chiu C-J , Hu J-C , Lo Y-H and Chang E-Y (2020) Health promotion and disease prevention interventions for the elderly: A scoping review from 2015-2019. International Journal of Environmental Research and Public Health 17(15), 1–11. 10.3390/ijerph17155335. PMC7432678 32722162

[ref15] Clancy A (2010) Perceptions of Public Health Nursing Practice: On Borders and Boundaries Visibility and Voice. [The Nordic School of Public Health], Sweden. Available at MicrosoftWord-RedigeringskopiA4_Rikke

[ref14] Clegg A , Young J , Iliffe S , Rikkert MO and Rockwood K (2013) Frailty in elderly people. Lancet 381(9868), 752–762. 10.1016/s0140-6736(12)62167-9 23395245 PMC4098658

[ref16] Clotworthy A (2017) Empowering the Elderly? A Qualitative Study of Municipal Home-Health Visits and Everyday Rehabilitation. [University of Copenhagen], Denmark. Available at https://researchprofiles.ku.dk/en/publications/empowering-the-elderly-a-qualitative-study-of-municipal-home-heal/

[ref17] Cowdell F , Dyson J , Sykes M , Dam R and Pendleton R (2022) How and how well have older people been engaged in healthcare intervention design, development or delivery using co-methodologies: A scoping review with narrative summary. Health & Social Care in the Community 30(2), 776–798. 10.1111/hsc.13199 33103313

[ref18] Cummins KM (2022) Explanations for the cloudy evidence that theory benefits health promotion. Frontiers in Psychology 13, 910041. 35846677 10.3389/fpsyg.2022.910041 PMC9285721

[ref19] Dahlgren S and Whitehead M (1991) *Policies and strategies to promote social equity in health*. Stockholm institute for further studies. Retrieved 15 October 2021 from https://core.ac.uk/download/pdf/6472456.pdf.

[ref21] Dengsø KE , Lindholm ST , Herling SF , Pedersen M , Nørskov KH , Collet MO , Nielsen IH , Christiansen MG , Engedal MS , Moen HW , Piil K , Egerod I , Hørder M and Jarden M (2023) Patient and public involvement in Nordic healthcare research: A scoping review of contemporary practice. Research Involvement and Engagement 9(1), 72. 10.1186/s40900-023-00490-x 37649111 PMC10466765

[ref22] Dinse D (2026) Group Exercises for Older Persons at Meeting Places in the Municipality: A Person-Centred Perspective. [Kristianstad University], Sweden. Avilable at https://researchportal.hkr.se/en/publications/group-exercises-for-older-persons-at-meeting-places-in-the-munici/

[ref23] Duplaga M , Grysztar M , Rodzinka M and Kopec A (2016) Scoping review of health promotion and disease prevention interventions addressed to elderly people. Bmc Health Services Research 16(Suppl 5), 455–465. 10.1186/s12913-016-1521-4 27608609 PMC5016725

[ref24] Eriksson A , Andersen HLM , Eriksson C , Johannessen A , Simonsen-Rehn N-H , Thualagant N , Torp S , Trollvik A and Haglund B (2020) How is health promotion research undertaken in Nordic context? A scoping review of doctoral dissertations from 2008-2018. Socialmedisinsk Tidskrift 97(3), 488–502.

[ref83] Eriksson K (Ed.) (1995) Den mångdimensionella hälsan: Verklighet och visioner. Institutionen för vårdvetenskap, Åbo Akademi.

[ref104] Eriksson M (2007) Unravelling the Mystery of Salutogenesis. Turku: Folkhälsan Research Centre.

[ref25] Eurostat (2020) *Ageing Europe: Looking at the lives of older people in the EU.* European Union. Retrieved 12 October 2021 from https://ec.europa.eu/eurostat/documents/3217494/11478057/KS-02-20-655-EN-N.pdf/9b09606c-d4e8-4c33-63d2-3b20d5c19c91?t=1604055531000.

[ref101] Fisher AG (2009) Occupational Therapy Intervention Process Model: A Model for Planning and Implementing, Top-Down, Client-Centered and Occupation Based Interventions. Fort Collins: Three Star Press.

[ref26] Fjell A (2021) *Preventive home visits among older people: Risk assessment, self-rated health, and experiences of healthy ageing*. [Karolinska Institutet], Sweden. Available at https://openarchive.ki.se/xmlui/handle/10616/47588.

[ref27] Forsman AK (2012) *The importance of social capital in later life mental health promotion and mental disorder prevention among older adults.* [Nordic School of Public Health], Sweden. Available at https://www.diva-portal.org/smash/get/diva2:1105141/FULLTEXT01.pdf.

[ref99] Gibson JJ (1986) The Ecological Approach to Visual Perception. New York: Routledge.

[ref111] Graham ID , Logan J , Harrison MB , Straus SE , Tetroe J , Caswell W and Robinson N (2006) Lost in knowledge translation: Time for a map? The Journal of Continuing Education in the Health Professions 26(1), 13–24. 10.1002/chp.47 16557505

[ref110] Green J (2015) Health Promotion: Planning & Strategies, 3rd Edn. Los Angeles: SAGE.

[ref28] Grundberg Å (2015) *Mental health promotion among community dwelling seniors with multimorbidity - perspectives of seniors, district nurses and home care assistants.* [Karolinska Institutet and Sophiahemmet University], Sweden. Available at https://openarchive.ki.se/xmlui/bitstream/handle/10616/44892/Thesis_%C3%85ke_Grundberg.pdf?sequence=1.

[ref29] Gustafsson S (2012) Health-Promoting Intervention for Community-Dwelling Older Adults. Focusing on the Concept of Frailty and Intervention Outcome. [University of Gothenburg], Sweden. Available at https://gupea.ub.gu.se/bitstream/handle/2077/28001/gupea_2077_28001_1.pdf?sequence=1.

[ref30] Haak M , Ivanoff S , Barenfeld E , Berge I and Lood Q (2021) Research as an essentiality beyond one’s own competence: An interview study on frail older people’s view of research. Research Involvement and Engagement 7(1), 91. 10.1186/s40900-021-00333-7 34952649 PMC8705152

[ref31] Haugan G and Eriksson M (2021) Health Promotion in Health Care – Vital Theories and Research. New York: Springer International Publishing. 36315659

[ref123] Holstein M and Minkler M (2007) Critical gerontology: Reflections for 21st century. In Bernard M and Scarf T (eds), Critical Perspectives on Ageing Society. Bristol: The Policy Press.

[ref91] Huber M , Knottnerus JA , Green L , van der Horst H , Jadad AR , Kromhout D , Leonard B , Lorig K , Loureiro MI , van der Meer JW , Schnabel P , Smith R , van Weel C and Smid H (2011) How should we define health? BMJ 343, Article d4163. 10.1136/bmj.d4163 21791490

[ref77] Iwarsson S , Edberg AK , Ivanoff SD , Hanson E , Jönson H and Schmidt S (2019) Understanding user involvement in research in aging and health. Gerontology & Geriatric Medicine 5, 2333721419897781. 10.1177/2333721419897781 31909093 PMC6937534

[ref92] Jans-Beken L , Jacobs N , Janssens M , Peeters S , Reijnders J , Lechner L and Lataster J (2019) Gratitude and health: An updated review. The Journal of Positive Psychology 15(6), 743–782.

[ref89] Janz NK and Becker MH (1984) The Health Belief Model: A decade later. Health Education Quarterly 11(1), 1–47. 10.1177/109019818401100101 6392204

[ref103] Jensen BB (2009) Et sundhedspædagogisk perspektiv på sundhedsfremme og forebyggelse. In Carlsson M, Simovska V and Jensen BB (eds), Sundhedspædagogik og sundhedsfremme. Århus: Århus Universitetsforlag, pp. 11–31.

[ref94] Kickbusch I (1992) Enhancing health potential. WHO Regional Publications, European Series (44), 8–10. 1515014

[ref78] Kickbusch I , Wait S and Maag D (2005) Navigating Health: The Role of Health Literacy. Alliance for Health and the Future, London, UK: International Longevity Centre.

[ref122] Kielhofner G (2008) Model of Human Occupation: Theory and Application, 4th Edn. USA: Lippincott Williams & Wilkins.

[ref32] Kleijberg M (2021) *Studio Döbra – Creating spaces for engaging with end-of-life issues and interacting across generations through community-based arts activities.* [Karolinska Institutet], Sweden. Available at https://openarchive.ki.se/xmlui/handle/10616/47553.

[ref33] Komulainen K (2013) *Oral health promotion among community-dwelling older people.* [University of Eastern Finland], Finland. Available at http://urn.fi/URN:ISBN:978-952-61-1213-8.

[ref84] Korp P (2016) Vad är hälsopromotion? Lund: Studentlitteratur.

[ref34] Koski J and Pihlainen K (2022) Participation of older people in learning studies: A scoping review. European Journal for Research on the Education and Learning of Adults 13(3), 285–299. 10.3384/rela.2000-7426.4188

[ref106] Krug EG, Dahlberg LL, Mercy JA, Zwi AB and Lozano R (eds). (2002) World Report On Violence And Health. Switzerland: World Health Organization.

[ref35] Kylén M , Slaug B , Jonsson O , Iwarsson S and Schmidt SM (2022) User involvement in ageing and health research: A survey of researchers’ and older adults’ perspectives. Health Research Policy and Systems 20(1), 93. 10.1186/s12961-022-00894-3 36050697 PMC9438331

[ref36] Laatikainen TE (2019) *Environments for healthy and active ageing.* [Aalto University], Finland. Available at https://aaltodoc.aalto.fi/items/91490a93-587a-4b1a-be0e-360acd765c9d.

[ref82] Lahtinen E, Lehtinen V, Riikonen E and Ahonen J (eds) (1999) Framework for Promoting Mental Health in Europe. Helsinki: National Research and Development Centre for Welfare and Health (STAKES).

[ref37] Larsson E (2016) *Promoting social activities and participation among seniors. Exploring and evaluating social and internet-based occupational therapy interventions.* [Umeå University], Sweden. Available at https://www.diva-portal.org/smash/get/diva2:895372/FULLTEXT01.pdf.

[ref100] Law M (2002) Participation in the occupations of everyday life. American Journal of Occupational Therapy 56(6), 640–649. 10.5014/ajot.56.6.640 12458856

[ref38] Liljas AEM , Walters K , Jovicic A , Iliffe S , Manthorpe J , Goodman C and Kharicha K (2017) Strategies to improve engagement of ‘hard to reach’ older people in research on health promotion: A systematic review. BMC Public Health 17(1), 1–12. 10.1186/s12889-017-4241-8 28431552 PMC5399821

[ref39] Ljungquist M (2018) *Caring deeds and habits – For a caritative approach to the act of caring* [Åbo Academy], Finland. Available at https://www.doria.fi/handle/10024/155512.

[ref41] Löfvendahl C (2025) Housing Policies to Promote the health of a population ageing in place: Comparisons using simulation modelling. [Lund University], Sweden. Available at https://lup.lub.lu.se/search/files/208267047/Avhandling_Christina_L_fvendahl_LUCRIS.pdf

[ref40] Lood Q (2015) Discovering the Capabilities of Ageing Persons who Are Born Abroad: Crossing Norms, Moving Health Promotion Forward. [University of Gothenburg], Sweden. Available at https://gupea.ub.gu.se/bitstream/handle/2077/37526/gupea_2077_37526_1.pdf.

[ref114] Lundman B , Viglund K , Aléx L , Jonsén E , Norberg A , Fischer RS , Strandberg G and Nygren B (2011) Development and psychometric properties of the Inner Strength Scale. International Journal of Nursing Studies 48(10), 1266–1274. 10.1016/j.ijnurstu.2011.03.006 21474137

[ref42] Mahler M (2012) *At holde balance. Betingelser for og perspektiver til forebyggelse af fald blandt gamle mennesker.* [Nordic School of Public Health], Sweden. Available at https://norden.diva-portal.org/smash/get/diva2:787178/FULLTEXT01.pdf.

[ref43] Malengreaux S , Doumont D , Scheen B , Van Durme T and Aujoulat I (2022) Realist evaluation of health promotion interventions: A scoping review. Health Promotion International 37(5), 1–15. 10.1093/heapro/daac136 36166263

[ref46] Mårtensson C (2012) *Promoting oral health. Knowledge of periodontal disease and satisfaction with dental care.* [Malmö University], Sweden. Available at https://www.diva-portal.org/smash/get/diva2:1404677/FULLTEXT01.pdf.

[ref44] McQueen DV , Kickbusch I , Potvin L , Pelikan JM , Balbo L and Abel T (2007) Health and Modernity: The Role of Theory in Health Promotion. New York: Springer Science & Business Media.

[ref45] Munn Z , Peters MDJ , Stern C , Tufanaru C , McArthur A and Aromataris E (2018) Systematic review or scoping review? Guidance for authors when choosing between a systematic or scoping review approach. BMC Medical Research Methodology 18(1), 143. 10.1186/s12874-018-0611-x 30453902 PMC6245623

[ref47] Nivestam A (2022) Health-Promoting Aspects of Preventive Home Visits for Older Persons: An Individual and Societal Perspective. [Lund University], Sweden. Available at https://portal.research.lu.se/en/publications/health-promoting-aspects-of-preventive-home-visits-for-older-pers/

[ref125] Nutbeam D (1998) Health promotion glossary. Health Promotion 13, 349–364. 10.1093/heapro/1.1.113 10318625

[ref79] Nutbeam D (2008) The evolving concept of health literacy. Social Science & Medicine 67(12), 2072–2078. 18952344 10.1016/j.socscimed.2008.09.050

[ref49] O’Brien KK , Colquhoun H , Levac D , Baxter L , Tricco AC , Straus S , Wickerson L , Nayar A , Moher D and O’Malley L (2016) Advancing scoping study methodology: A web-based survey and consultation of perceptions on terminology, definition and methodological steps. BMC Health Services Research 16(1), 305. 10.1186/s12913-016-1579-z 27461419 PMC4962390

[ref50] Olofsson N (2012) Violence through the Life Cycle: A Public Health Problem. [Linköping University], Sweden. Available at https://liu.diva-portal.org/smash/get/diva2:524716/FULLTEXT01.pdf

[ref108] Orem DE (2001) Nursing: Concepts of Practice, 6th Edn. St. Louis: Mosby-Year Book.

[ref51] Pedersen MK , Beck AM , Boateng T , Brorholt G and Overgaard D (2022) Patient and public involvement – Erfaringer fra et dansk peer-mentor interventions projekt. Nordisk Sygeplejeforskning 12(2), 1–13. 10.18261/nsf.12.2.6

[ref52] Peters M , Godfrey C , McInerney P , Munn Z , Tricco A and Khalil H (2020) Scoping reviews. In Aromataris E and Munn Z (eds), JBI Manual for Evidence Synthesis. Australia: Joanna Briggs Institute, Australia, pp. 407–452. 10.46658/JBIMES-20-12. https://synthesismanual.jbi.global

[ref53] Pii KH , Schou LH , Piil K and Jarden M (2019) Current trends in patient and public involvement in cancer research: A systematic review. Health Expectations 22(1), 3–20. 10.1111/hex.12841 30378234 PMC6351419

[ref113] Prochaska JO and DiClemente CC (1983) Stages and processes of self-change of smoking: Toward an integrative model of change. Journal of Consulting and Clinical Psychology 51(3), 390–395. 10.1037/0022-006X.51.3.390 6863699

[ref119] Putnam RD (1993) Making Democracy Work. Civic Traditions in Modern Italy. Princeton: Princeton University Press.

[ref54] Pyae A (2020) The use of digital games to enhance the physical exercise activity of the elderly: A case of Finland. [University of Turku], Finland. Available at https://www.utupub.fi/handle/10024/148945.

[ref128] Regjerinenno. (2023). Folkehelsemeldingen nr. 15, Nasjonal Strategi for utjevning av sosiale helseforskjeller. Public Health Report Meld. St. 15 (2022-2023to the Storting (White Paper) https://www.regjeringen.no/no/dokumenter/meld.-st.-15-20222023/id2969572/

[ref56] Rootman I (2001) Evaluation in Health Promotion: Principles and Perspectives. Denmark: World Health Organization, Regional Office for Europe.

[ref57] Rootman I , Goodstadt M , Potvin L and Springett J (2001) A framework for health promotion evaluation. WHO Regional Publications, European Series (92), 7–38. 11729789

[ref76] Sahlen KG , Löfgren C , Brodin H , Dahlgren L and Lindholm L (2012) Measuring the value of older people’s production: a diary study. BMC Health Services Research 12, Article 4. 10.1186/1472-6963-12-4 PMC3295658 22230745

[ref81] Sallis J , Cervero RB , Ascher W , Henderson KA , Kraft MK and Kerr J (2006) An ecological approach to creating active living communities. Annual Review of Public Health 27, 297–322. 10.1146/annurev.publhealth.27.021405.102100 16533119

[ref95] Sallis J and Owen N (2015) Ecological models of health behavior. In Glanz K, Rimer BK and Viswanath K (eds), Health Behavior: Theory, Research, and Practice, 5th Edn. San Francisco: Jossey-Bass, pp. 43–64.

[ref85] Sallis JF and Neville O (2015) Ecological models of health behavior. In Glanz K, Rimer BK and Viswanath K (eds), Health Behavior: Theory, Research, and Practice, 5th Edn. San Francisco: Jossey-Bass A Wiley Imprint, pp. 465–485.

[ref88] Sen A (1993) Capability and well-being. In Nussbaum M and Sen A (eds), The Quality of Life. Oxford: Clarendon Press, pp. 30–53.

[ref124] Settersten RA jr (2003) Invitation to Life Course; Toward New Understandings of Later Life. Amityville: Baywood Publishing Company.

[ref58] Shippee ND , Domecq Garces JP , Prutsky Lopez GJ , Wang Z , Elraiyah TA , Nabhan M , Brito JP , Boehmer K , Hasan R , Firwana B , Erwin PJ , Montori VM and Murad MH (2015) Patient and service user engagement in research: A systematic review and synthesized framework. Health Expectations 18(5), 1151–1166. 10.1111/hex.12090 23731468 PMC5060820

[ref59] Sjöblom M (2020) *Health promotion through the lifespan - The phenomenon of the inner child reflected in childhood events experienced by children, adults and older persons* [Luleå University of technology], Sweden. Available at https://www.diva-portal.org/smash/get/diva2:1370318/FULLTEXT01.pdf.

[ref127] Söderhamn O (1998) Self-Care ability in a group of elderly Swedish people: a phenomenological study. Journal of Advanced Nursing 28(4), 745–753. 9829662 10.1111/j.1365-2648.1998x.00705.x

[ref60] SOU Statens offentliga utredrningar (2020) God och nära vård: En reform för ett hållbart hälso-och sjukvårdssystem. Retrieved 10 October 2024 from https://www.regeringen.se/contentassets/320f37078d854712ab89e8185466817b/god-och-nara-vard-en-reform-for-ett-hallbart-halso--och-sjukvardssystem-sou_2020_19_webb.pdf.

[ref97] Stokols D (1992) Establishing and maintaining healthy environments: toward a social ecology of health promotion. American Psychologist 47(1), 6. 1539925 10.1037//0003-066x.47.1.6

[ref98] Stokols D (1996) Translating social ecological theory into guidelines for community health promotion. American Journal of Health Promotion 10(4), 282–298. 10159709 10.4278/0890-1171-10.4.282

[ref20] Sundhedsstyrelsen (2024) Sundhedsfremme og sund aldring. Retrieved 10 September 2023 from www.sst.dk.

[ref61] Sundsli K (2015) *Engagement, knowledge, and autonomy – Facing a new generation older urban living people: studies on self-care and health.* [Norwegian University of Science and Technology], Norway. Available at https://ntnuopen.ntnu.no/ntnu-xmlui/handle/11250/283323.

[ref48] Suominen S (2014) Nordic school of public health NHV 60 years of public health. Nordiska ministerrådet: Nordic School of Public Health. Available at https://norden.diva-portal.org/smash/get/diva2:787735/FULLTEXT02.pdf.

[ref62] Thualagant N , Simonsen N , Sarvimäki A , Stenbock-Hult B , Olafsdottir HS , Fosse E , Torp S , Ringsberg KC , Forrinder U and Tillgren P (2023) Nordic responses to covid-19 from a health promotion perspective. Health Promotion International 38(4), 1–13. 10.1093/heapro/daab211 PMC8807276 35022714

[ref64] Tøien M (2019) An exploration of how long-term preventive home visits affect older persons’ health and possibility for a good life in their own homes. Users’ and service-providers’ perspectives [University of Oslo], Norway. Available at https://www.duo.uio.no/bitstream/handle/10852/67942/PhD-Toien-DUO.pdf?sequence=1&isAllowed=y.

[ref117] Tones K and Green J (2004) Health Promotion: Planning and Strategies. London: Sage.

[ref63] Tricco AC , Lillie E , Zarin W , O’Brien KK , Colquhoun H , Levac D , Moher D , Peters MDJ , Horsley T , Weeks L , Hempel S , Akl EA , Chang C , McGowan J , Stewart L , Hartling L , Aldcroft A , Wilson MG , Garritty C , Lewin S , Godfrey CM , Macdonald M T , Langlois EV , Soares-Weiser K , Moriarty J , Clifford T , Tunçalp Ö and Straus SE (2018) PRISMA extension for scoping reviews (PRISMA-ScR): Checklist and explanation. Annals of Internal Medicine 169(7), 467–473. 30178033 10.7326/M18-0850

[ref121] Utz RL , Carr D , Nesse R and Wortman CB (2002) The effect of widowhood on older adults’ social participation: An evaluation of activity, disengagement, and continuity theories. The Gerontologist 42(4), 522–533. 10.1093/geront/42.4.522 12145380

[ref65] Vercauteren T , Van Regenmortel S , Näsman M , Nyqvist F , Brosens D , serrat R and Dury S (2024) Multi-dimensional civic engagement of older Europeans: A latent class analysis. Ageing & Society 45(10), 1997–2022. 10.1017/S0144686X2400062X

[ref66] Vorup J (2017) Health and physiological adaptations of small sided ball games in untrained older adults aged 65–93 years, [University of Copenhagen], Denmark. Retrieved 10 September 2023 from: https://www.ucviden.dk/ws/portalfiles/portal/187626260/PhD_thesis_Jacob_Vorup.pdf.

[ref67] Wallerstein NB and Duran B (2006) Using community-based participatory research to address health disparities. Health Promotion Practice 7(3), 312–323. 10.1177/1524839906289376. 16760238

[ref68] Wennerberg M (2017) *Unravelling the duality of caregivinghood - how informal caregivers describe their situation when salutogenically approached*, [University of Gothenburg], Sweden. Available at https://gupea.ub.gu.se/handle/2077/51880.

[ref70] WHO (1986) The Ottawa Charter for Health Promotion, 1986. Canada: World Health Organization. Retrieved 15 October 2021 from https://www.euro.who.int/__data/assets/pdf_file/0004/129532/Ottawa_Charter.pdf.

[ref120] WHO (2004) A Glossary of Terms for Community Health Care and Services for Older Persons. Japan: WHO.

[ref80] WHO (2007) Constitution of the World Health Organization. Retrieved from http://apps.who.int/gb/bd/PDF/bd47/EN/constitution-en.pdf

[ref93] WHO (2014) Mental health: Strengthening our response. https://www.who.int/mediacentre/factsheets/fs220/en/

[ref116] WHO (2015) World Report on Ageing and Health. Geneva: WHO. Retrieved 10 October 2024 from: https://www.who.int/ageing/events/world-report-2015-launch/en/

[ref69] WHO (2018) A Vision for Primary Health Care in the 21st Century: Towards Universal Health Coverage and the Sustainable Development Goals. Geneva: World Health Organization and the United Nations Children’s Fund (UNICEF). Retrieved 14 January 2026 from https://www.who.int/docs/default-source/primary-health/vision.pdf.

[ref71] WHO (2021) Healthy Ageing and the Sustainable Development Goals. World Health Organization. Retrieved 1 June 2021 from https://www.who.int/ageing/sdgs/en/.

[ref72] Wiklund-Axelsson S (2015) *Prerequisites for sustainable life style changes among older persons with obesity and for ict support* [University of Technology, Luleå], Sweden. Available at https://www.diva-portal.org/smash/get/diva2:989795/FULLTEXT01.pdf.

[ref102] Wilcock AA (1993) A theory of the human need for occupation. Journal of Occupational Science 1(1), 17–24. 10.1080/14427591.1993.9686375

[ref112] Wilcock AA (1998) Occupation for health. British Journal of Occupational Therapy 61(8), 340–345.

[ref73] Woodall J and Freeman C (2020) Where have we been and where are we going? The state of contemporary health promotion. Health Education Journal 79(6), 621–632. 10.1177/0017896919899970

[ref74] Woodall J , Warwick-Booth L , South J and Cross R (2018) What makes health promotion research distinct? Scandinavian Journal of Public Health 46(20_suppl), 118–122. 10.1177/1403494817744130 29552972

[ref75] Zingmark M (2015) *Occupation-focused and occupationbased interventions for communitydwelling older people intervention effects in relation to facets of occupational engagement and cost effectiveness*, [Umeå University], Sweden. Available at https://umu.diva-portal.org/smash/get/diva2:789740/FULLTEXT01.pdf.

